# Electromagnetic wireless remote control of mammalian transgene expression

**DOI:** 10.1038/s41565-025-01929-w

**Published:** 2025-05-05

**Authors:** Zhihua Lin, Preetam Guha Ray, Jinbo Huang, Peter Buchmann, Martin Fussenegger

**Affiliations:** 1 Department of Biosystems Science and Engineering, ETH Zurich, Basel, Switzerland; 2 University of Basel, Faculty of Life Science, Basel, Switzerland

**Keywords:** Biomaterials, Biosensors, Bionanoelectronics, Nanoparticles

## Abstract

Communication between wireless field receivers and biological sensors remains a key constraint in the development of wireless electronic devices for minimally invasive medical monitoring and biomedical applications involving gene and cell therapies. Here we describe a nanoparticle–cell interface that enables electromagnetic programming of wireless expression regulation (EMPOWER) of transgenes via the generation of cellular reactive oxygen species (ROS) at a biosafe level. Multiferroic nanoparticles coated with chitosan to improve biocompatibility generate ROS in the cytoplasm of cells in response to a low-frequency (1-kHz) magnetic field. Overexpressed ROS-responsive KEAP1/NRF2 biosensors detect the generated ROS which is rewired to synthetic ROS-responsive promoters to drive transgene expression. In a proof-of-concept study, subcutaneously implanted alginate-microencapsulated cells stably expressing an EMPOWER-controlled insulin expression system normalized blood-glucose levels in a mouse model of type 1 diabetes in response to a weak magnetic field.

## Abstract (web summary)

Wireless magnetic control of gene expression in mammalian cells has been developed based on intracellular nanointerface and ROS-mediated signalling. The approach allows remotely tunable insulin release and regulates blood glucose in diabetic mice.

## Main

Synthetic biology has revolutionized cell engineering for alleviating numerous diseases^1,2^, including chronic pain^3^, obesity^4^, diabetes^5^, cancer^6^ and muscle atrophy^7^, and for investigating neural circuits^8^ and bioelectronics interfaces^9,10^. In particular, physical stimuli of gene circuits, such as light^11^, sound^12^, electrical signals^13,14^ and magnetic fields^15,16^, have been intensively explored for spatiotemporal control of therapeutic outputs. To circumvent the challenge of wireless signal propagation, electromagnetic fields (EMFs) of varying strength, frequency, duration and location have been exploited in conjunction with the mechanical^17–19^ or thermal properties^8,20,21^ of magnetic nanoparticles to enable coupling with ion channels on cell membranes. EMFs with an amplitude of <50 mT and a frequency of <1 MHz minimize energy dissipation in living tissues^22^, and therefore are suitable for remotely programmable switches to stimulate cellular functions with minimal influence on native systems. However, legacy technology pioneering the electromagnetic programming of cellular behaviour was based on cell-specific in vivo coordination of inorganic nanoparticles to channels or receptors of native or engineered cells using antibodies^23,24^ or tags^16,18,25^, which may elicit off-target effects of conjugated nanoparticles^26,27^, promote liver toxicity^20,28^ or limit robustness due to intracellular trafficking of channels and receptors^29^, resulting in limited tunability^22^ and biosafety^30^. We have therefore designed and tested a versatile and robust genetic interface enabling tunable remote control of therapeutic transgene expression by microencapsulated designer cells using low-power EMF.

Multiferroic materials that harmonize magnetostrictive and piezoelectric effects can exploit magnetic fields to generate electricity for biological applications, such as remote brain activity detection, deep neural stimulation^31^, bone defect repair^32^ and degradation of Alzheimer’s β-amyloid aggregates^33^. These effects occur as aqueous solvents and solutes transfer charge carriers from multiferroic material surfaces to produce electrophiles, mostly reactive oxygen species (ROS)^34,35^. Thus, a.c. millitesla EMFs in the low-frequency range (0.1–1 kHz) hold promise as a biological portal via ROS.

ROS act as native cytoplasmic signals in living systems, and human cells contain components that can sense and respond to them^36,37^. When exposed to elevated ROS, Kelch-like ECH-associated protein 1 (KEAP1), which contains ROS-sensitive cysteine residues, releases nuclear factor erythroid 2 p45-related factor 2 (NRF2), allowing NRF2 to translocate and bind to intranuclear antioxidant-response elements (AREs), resulting in transcriptional antioxidant responses^38^. Here we utilized magnetoresponsive ROS-generating multiferroic (CoFe_2_O_4_@BiFeO_3_@chitosan, CBCFO) nanoparticles to communicate with cells sensitized to ROS by overexpressing KEAP1/NRF2 and rewired NRF2 to synthetic ARE-containing promoters, thereby constructing a system that we term electromagnetic programming of wireless expression regulation (EMPOWER). In this system, the embedded CBCFO nanoparticles serve as nanoreceivers of an external electromagnetic field, providing electromagnetic tunability of ROS generation to drive transgene expression of the target protein by the host cells. For proof of concept and as an example, we chose to validate the EMPOWER system for blood-glucose management in experimental type 1 diabetes (T1D) because diabetes is a dynamically highly challenging medical condition with dramatically increasing prevalence^39–41^. Therefore, we implanted transgenic human cells with the EMPOWER system enclosed in coherent, clinically licensed alginate microcapsules into T1D mice and exposed them to an EMF to control insulin release. Low-frequency EMF (1 kHz) stimulation of 21 mT for 3 min per day effectively induced insulin secretion from the subcutaneously implanted EMPOWER-controlled designer cells and restored normoglycaemia in T1D mice over the entire 4-week experimental period.

## Results

### Characterization of chitosan-multiferroic nanoparticles

**Fig. 1:**
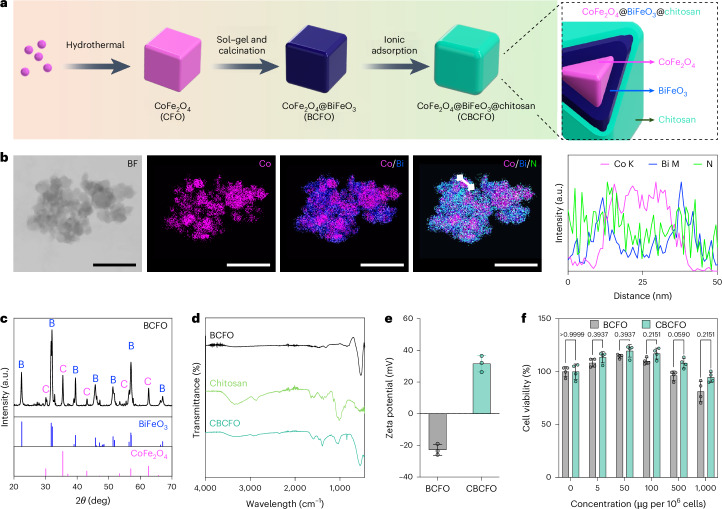
Construction and characterization of CBCFO nanoparticles. **a**, Illustration of the synthesis of CBCFO nanoparticles. **b**, STEM bright-field (BF) images and corresponding EDX results with colocalized elemental mapping of cobalt, bismuth and nitrogen are consistent with a core–shell structure of CBCFO nanoparticles. Scale bar, 50 nm. **c**, XRD pattern displaying the crystallinity of BCFO. Black, BCFO; B, peaks from BiFeO_3_; C, peaks from CoFe_2_O_4_. **d**, Attenuated total reflectance infrared spectra of BCFO, chitosan and CBCFO. **e**, Zeta potential of BCFO and CBCFO, before and after coating with the chitosan layer. **f**, Cell viability upon exposure to CBCFO nanoparticles is dependent on nanoparticle mass per million cells. Data are presented as mean ± s.d., *n* = 3 (**e**) or *n* = 4 (**f**) independent experiments. *P* values in **f** were calculated versus the corresponding non-induced control by a two-sided unpaired *t*-test. Source data

To sense the magnetic field for ROS-mediated transgene expression control, we synthesized core–shell CoFe_2_O_4_@BiFeO_3_ (BCFO) multiferroic nanoparticles consisting of magnetostrictive CoFe_2_O_4_ (CFO) nanoparticle cores and piezoelectric BiFeO_3_ (BFO) shells, with the chitosan outer layer to form the CBCFO nanoparticles, as illustrated in Fig. 1a. Scanning transmission electron microscopy (STEM) imaging and corresponding energy-dispersive X-ray spectroscopy (EDX) mapping (Fig. 1b) confirmed the structure of the CBCFO nanoparticles, as demonstrated by the distributions of cobalt, bismuth and nitrogen in the CFO, BFO and chitosan. The line profile of the CBCFO nanoparticle shows the representative spatial distributions of cobalt, bismuth and nitrogen, quantitatively confirming the structure. The EDX spectrum indicated similar atom contents of cobalt and bismuth in the CBCFO nanoparticles (Supplementary Fig. 1), in contrast with CFO (Supplementary Fig. 2a) and BCFO (Supplementary Fig. 2b) nanoparticles. The CBCFO nanoparticles were 35.5 ± 10.3 nm in diameter, while the diameters of CFO and BCFO nanoparticles were 25.4 ± 6.1 nm and 32.9 ± 8.5 nm, respectively, according to the transmission electron microscopy (TEM) results (*n* = 50 particles). At the physiological pH of 7.4, the hydrodynamic diameter of CBCFO nanoparticles is 36.3 ± 4.8 nm and their polydispersity index reaches 0.190 (Supplementary Fig. 3). The X-ray diffraction (XRD) patterns revealed the cubic spinel structure of CFO with an *Fd*3*m* space group, and the rhombohedral perovskite structure of BFO with an *R*3*c* space group (Fig. 1c)^33,42^.

**Extended Data Fig. 1:**
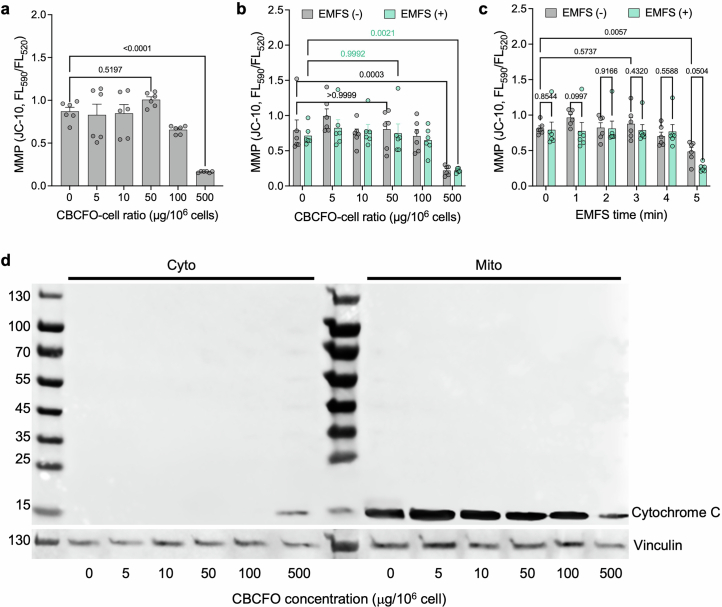
Impact of CBCFO nanoparticle concentration and EMFS on mitochondrial membrane potential (MMP) and cytochrome C release. (**a**) Relationship between MMP and CBCFO nanoparticle concentration 24 h after addition. (**b**) CBCFO nanoparticle concentration-dependent MMP 24 h after EMF stimulation (21 mT, 1 kHz, 3 min). (**c**) EMFS time-dependent MMP by CBCFO nanoparticles (50 µg/10^6^ cells) 24 h after EMFS (21 mT, 1 kHz, 0–5 min). (**d**) Western blot-based analysis of cytochrome C release from mitochondria 24 h after addition of different concentrations of CBCFO nanoparticles (0–500 µg/10^6^ cells). Vinculin was used as a loading control. All data are presented as means ± s.d.; n = 6 independent experiments. Statistical significance was analysed by one-way ANOVA with Tukey’s multiple comparisons test in (**a**) and two-way ANOVA with Dunnett’s multiple comparisons test in (**b, c**). Source data

In attenuated total reflectance infrared analysis, the region between 800 and 1,200 cm^−1^ shows characteristic absorption of chitosan saccharide structure (Fig. 1d)^43^. The chitosan protonation and hydration processes during coating of CBCFO nanoparticles are reflected in changes in the asymmetric –NH band between 1,300 and 1,700 cm^−1^ compared with chitosan powder. The resulting ammonium groups within the chitosan layer of CBCFO nanoparticles contribute to the positive surface charge of 31.6 ± 4.6 mV compared with the negative charge (−22.5 ± 5.5 mV) of BCFO nanoparticles (Fig. 1e and Supplementary Fig. 4). Cells containing up to 50 μg BCFO or 100 μg CBCFO per 10^6^ human embryonic kidney cells (HEK-293) retained more than 95% viability after 48 h (Fig. 1f). These results guided our choice of concentration range for the following in vitro evaluation. The decrease in cell viability at higher CBCFO concentrations was directly correlated with cytosolic accumulation, and presumably resulted from excessive changes in mitochondrial membrane potential which triggered apoptosis-associated release of cytochrome C (Extended Data Fig. 1).

### Electromagnetically induced ROS production in vitro

**Fig. 2:**
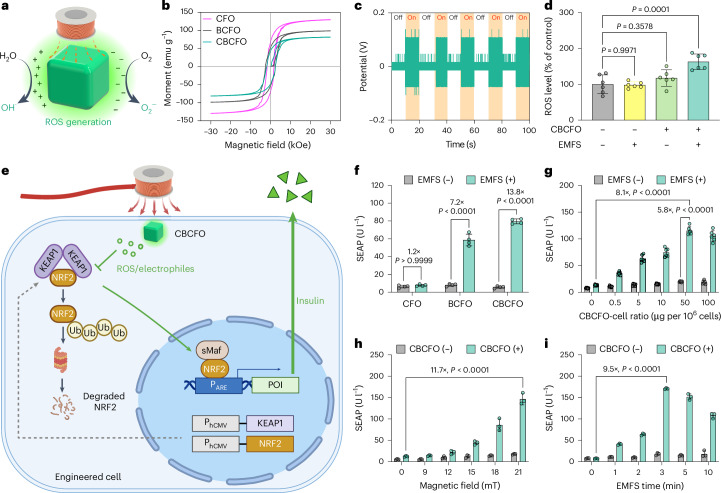
Electromagnetically stimulated ROS production and transgene expression in transiently transfected cells. **a**, Schematic illustration of the magnetoelectric effect of CBCFO nanoparticles and ROS production. **b**, Magnetic hysteresis curves of CFO, BCFO and CBCFO at r.t. with magnetic field strengths ranging from −30 kOe to +30 kOe. **c**, The on/off behaviour of the OCV induced by CBCFO nanoparticles depends on the applied a.c. magnetic field (21 mT, 1 kHz). **d**, Fluorescence-based quantification of cellular ROS levels after EMF stimulation (21 mT, 1 kHz, 3 min). **e**, Scheme of the proposed mechanism of electromagnetically induced gene expression in engineered responsive cells transfected with pJH1003, pJH1004 and pJH1005. The ROS generated by EMF-stimulated CBCFO disrupts the interaction between KEAP1 and NRF2, thereby inhibiting ubiquitination by the KEAP1-associated ubiquitin (Ub) ligase complex. Consequently, NRF2 translocates to the nucleus, where it binds to small Maf proteins (sMaf) and to the antioxidant-response elements (ARE) in the regulatory regions of its target genes. **f**, SEAP production by transiently transfected ROS-responsive cells containing CFO, BCFO and CBCFO nanoparticles. **g**, SEAP expression is dependent on the CBCFO–cell ratio (magnetic field, 21 mT; 3 min). **h**, Electromagnetic-field-dependent gene expression (stimulation time, 3 min). SEAP expression was maximum at 21 mT, reaching peak levels that compare to SEAP levels of isogenic cells in which SEAP is driven by a strong constitutive promoter (149.3 ± 8.7 U l^−1^). **i**, Stimulation-time-dependent gene expression (magnetic field, 21 mT). Data are presented as mean ± s.d., *n* = 6 (**d**,**g**), *n* = 4 (**f**) or *n* = 3 (**h**,**i**) independent experiments. *P* values in **d**–**i** were calculated versus the corresponding non-stimulated control. Statistical significance was analysed by one-way ANOVA with Dunnett’s multiple-comparisons test (**d**), two-way ANOVA with Bonferroni’s multiple comparisons test (**f**) and two-way ANOVA with Dunnett’s multiple comparisons test (**g**,**h**,**i**). Mechanism schematics created with BioRender.com. Source data

**Extended Data Fig. 2:**
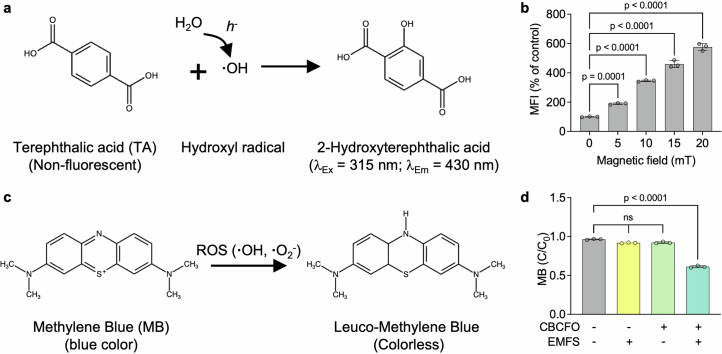
Quantification of charge carriers and ROS. (**a**) The mechanism of TA assay to detect charge carriers. (**b**) The mean fluorescence intensities (MFI) show the magnetic-field-dependent generation of 2-hydroxyterephthalic acid. (**c**) The mechanism of MB assay to detect ROS production in the aqua-based environment. (**d**) Decrease of MB induced by CBCFO and EMFS, measured in terms of the absorbance at 664 nm. All data are presented as means ± s.d.; (**b, d**) n = 3 independent experiments. Statistical significance was analysed by one-way ANOVA with Tukey’s multiple comparisons test in (**b, d**). Source data

Under a.c. electromagnetic field stimulation (EMFS), multiferroic BCFO generates electric polarization due to the interfacial lattice strain between BFO and CFO^44,45^. Charge separation of BCFO affords excited charge carriers on the surface of CBCFO nanoparticles, leading to local production of ROS such as superoxide radical (O_2_^−·^) and hydroxyl radical (OH^·^) in an aqueous environment^33^ (Fig. 2a). CBCFO nanoparticles exhibit magnetic hysteresis loops (EMF range, −30 to 30 kOe) under ambient conditions (Fig. 2b), with a saturation magnetization (*M*_s_) and remnant magnetization (*M*_r_) of 77.8 and 45.2 emu g^−1^, respectively, signifying room-temperature ferromagnetism. The decreased ferromagnetism of CBCFO derived from BCFO nanoparticles (*M*_s_ = 96.8 emu g^−1^, *M*_r_ = 57.2 emu g^−1^) is attributable to the content of chitosan. For the following experiments, we employed EMFs of 1 kHz frequency and up to 21 mT field strength to avoid any adverse thermal effect^22^ in living systems and to maintain effective coupling of the BFO–CFO interface^44^. A Helmholtz-coil-based device was assembled to generate a uniform a.c. EMF of 9–21 mT in multiwell plates (Supplementary Fig. 5). The induced electrical potential of CBCFO powder in an open-circuit-voltage (OCV) set-up (Supplementary Fig. 6a) was measured with an EMF of 1 kHz and 21 mT and reached 0.11 V (Fig. 2c), in contrast with the device bias control of 0.016 V (Supplementary Fig. 6b). The relative charge separation was detected by a terephthalic acid (TA) assay depending on the EMFS strength (Extended Data Fig. 2a,b). The capability of the charge carriers to induce ROS was evaluated by measuring the non-specific ROS-mediated decolorization of methylene blue (MB assay, Extended Data Fig. 2c,d). The degradation rate of 39% with CBCFO nanoparticles (5 mg ml^−1^) after 1-h EMFS (1 kHz, 21 mT) indicates a significant ROS production from CBCFO with EMFS, compared with bare CBCFO and EMFS-alone control groups.

**Extended Data Fig. 3:**
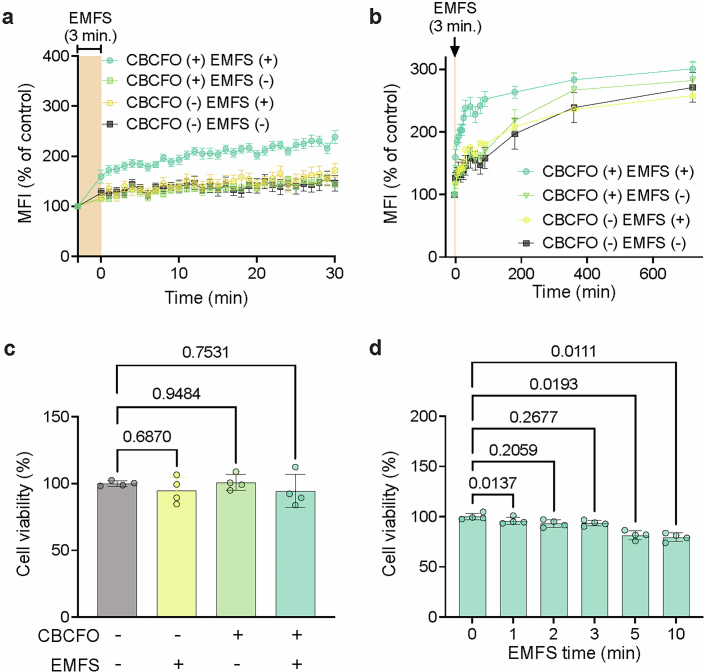
In vitro ROS production stimulated by the magnetic field. (**a**) Significantly increased production of ROS over 30 min in HEK-293 cells with CBCFO after EMFS (1 kHz, 21 mT, 3 min). (**b**) Accumulated ROS in the cells reached a maximum at about 6 h after stimulation in (**a**). All groups show similar increases in ROS production at 12 h after stimulation. Percent mean fluorescence intensity (MFI %) represents the mean fluorescence intensity normalized to blank assay fluorescence. (**c**) Cell viability 72 h after EMF stimulation. (**d**) EMF stimulation-time-dependent cell viability (21 mT, 1 kHz, 0–10 min). All data are presented as means ± s.d.; n = 4 independent experiments. Statistical significance was analysed by one-way ANOVA with Dunnett’s multiple comparisons test in (**c, d**). Source data

In vitro quantification showed that intracellular ROS production was accelerated with CBCFO in contrast to control groups immediately after 3 min EMFS (1 kHz, 21 mT) (Fig. 2d). The acceleration occurred mostly within the first 30 min (Extended Data Fig. 3a) and declined in the following 3–6 h (Extended Data Fig. 3b). We confirmed no significant difference in cell viability among stimulated and non-stimulated groups due to this accelerated ROS production (Extended Data Fig. 3c), and cell viability started to decrease with only EMFS of 5 min or longer (Extended Data Fig. 3d).

### Electromagnetically controlled transient gene expression

**Extended Data Fig. 4:**
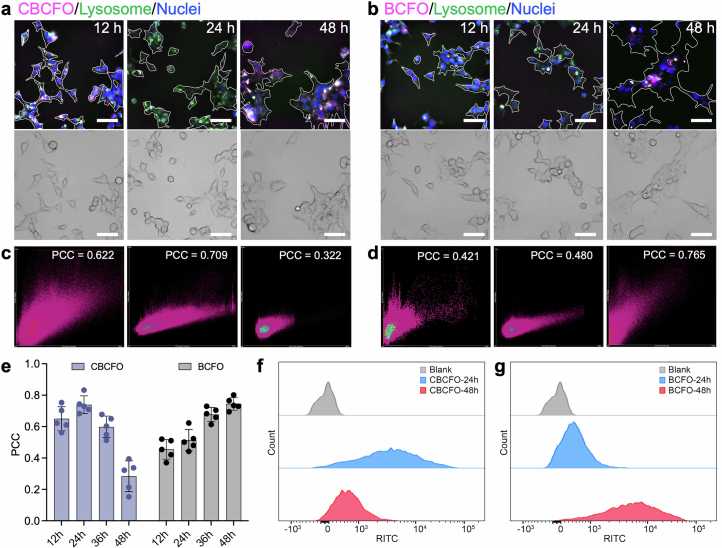
Endosome escape behaviour. Representative fluorescence microscopy images of HEK-293 cells incubated with RITC (Ex/Em: 531/593, red)-labelled (**a**) CBCFO and (**b**) BCFO nanoparticles for 12, 24 and 48 h. For co-localization analysis, lysosomes (Lyso tracker green, Ex/Em: 466/495, green) and nuclei (Hochst-33342, Ex/Em: 387/409, blue) were labelled. The region of interest (ROI, white) was derived from the bright-field images. Pearson’s correlation coefficient (PCC) was calculated from the co-localization results between lysosomes and (**c**) CBCFO, (**d**) BCFO nanoparticles. Statistical quantification (**e**) shows enhanced endosome escape of CBCFO nanoparticles from the lysosomes. Flow-cytometric analysis of cells incubated with (**f**) CBCFO and (**g**) BCFO nanoparticles confirmed the endosome escape behaviour. In all these experiments, CBCFO nanoparticles are applied as 50 μg/10^6^ cells. All data are presented as means ± s.d.; n = 5 independent experiments. Source data

**Extended Data Fig. 5:**
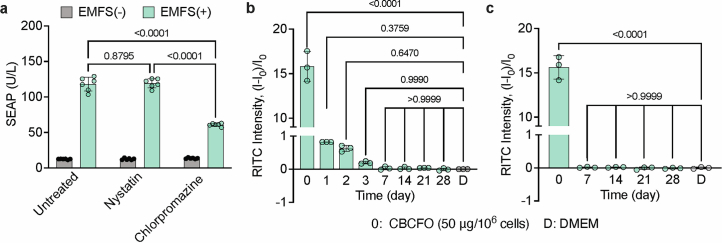
Endocytosis and cellular extrusion of CBCFO nanoparticles. (**a**) Impact of CBCFO nanoparticle endocytosis inhibition on EMF-stimulated SEAP expression. Inhibition of classical clathrin-mediated endocytosis by chlorpromazine decreases endocytosis-mediated uptake of CBCFO nanoparticles, reduces EMF-triggered ROS-production and attenuates ROS-induced SEAP expression. (**b, c**) Fluorescence-based extrusion of rhodamine B isothiocyanate-labelled CBCFO nanoparticles (50 mg/10^6^ cell) from native (**b**) and microencapsulated (**c**) HEK_EMPOWER_ cells. Fluorescence intensity (I) (**b**, **c**) was normalized to DMEM fluorescence (I_0_). Data are presented as means ± s.d.; n = 5 (**a**); n = 3 (**b**, **c**) independent experiments. Statistical significance was analysed by two-way ANOVA with Dunnett’s test. Source data

**Extended Data Fig. 6:**
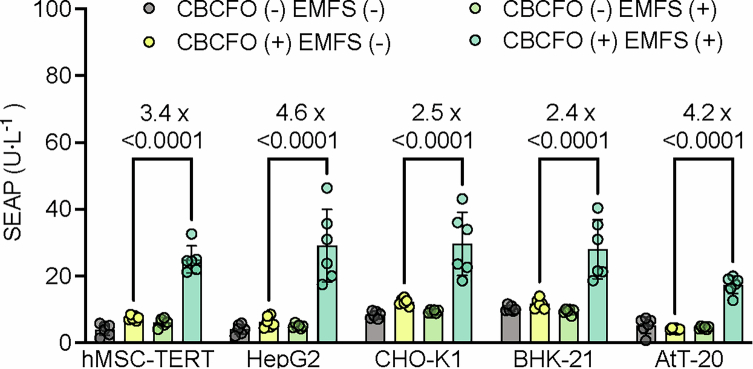
EMF-controlled SEAP expression in various mammalian cell types. 10^4^ hMSC-TERT (human), HepG2 (human), CHO-K1(hamster), BHK-21 (hamster), and AtT-20 (mouse) cells were co-transfected with EMPOWER vectors pJH1003, pJH1004 and pJH1005, loaded with CBCFO nanoparticles (50 μg/10^6^ cells) and optionally stimulated by EMF (21 mT, 1 kHz, 3 min). All data are presented as means ± s.d.; n = 6 independent experiments. Statistical significance was calculated by two-way ANOVA with Dunnett’s multiple comparisons test. Source data

To utilize EMF for gene expression, we cotransfected HEK-293 cells with constitutive *KEAP1* (pJH1004, *P*_*hCMV*_*-KEAP1-pA*) and *NRF2* (pJH1003, *P*_*hCMV*_*-NRF2-pA*) expression plasmids to construct a ROS-biosensing system, together with the reporter pJH1005 (*P*_*DART*_*-SEAP-pA*; *P*_*DART*_, *O*_*ARE*_*-P*_*hCMVmin*_) encoding the model human glycoprotein SEAP (human placental secreted alkaline phosphatase) for quantification of the expression level (Supplementary Table 1). In these cells, electromagnetically induced cellular ROS production via CBCFO nanoparticles interferes with the NRF2–KEAP1 interaction, leading to release and translocation of NRF2 to the nucleus, which results in expression of the protein of interest (POI) from the NRF2-specific ARE-containing P_DART_ promoter (Fig. 2e). In comparison with CFO-embedded engineered cells, electromagnetically stimulated SEAP expression was significantly elevated (13.8-fold) compared with the non-stimulated control (Fig. 2f). The CBCFO group afforded lower leakiness and a higher expression level than the BCFO group, in accordance with the higher cellular uptake and improved endosome-escape capability as judged from time-lapse microscopy images (Extended Data Fig. 4a,b), fluorescence colocalization (Extended Data Fig. 4c–e) and flow cytometry (Extended Data Fig. 4f,g). These results can be attributed to the proton sponge effect of chitosan modification^46^. Cellular uptake of CBCFO nanoparticles occurs via classical clathrin-mediated endocytosis (Extended Data Fig. 5a) and no cellular nanoparticle extrusion occurred beyond 3 days after cellular uptake (Extended Data Fig. 5b). Leakage was not observed from implant preparations in 4 weeks, confirming the integrity of the alginate-based microcapsules (Extended Data Fig. 5c). The transgene expression level increased with increasing concentration of CBCFO, peaking at a CBCFO concentration of 50 μg per 10^6^ cells under an EMF of 21 mT and 1 kHz for 3 min (Fig. 2g), corresponding to 1.5 × 10^4^ J s m^−3^ in volume-averaged energy density. At the CBCFO concentration of 50 μg per 10^6^ cells, the SEAP level increased along with EMF strength (0–21 mT; Fig. 2h). The expression level of SEAP could be precisely adjusted by varying the EMFS time at 21 mT (Fig. 2i). The EMPOWER is characterized in HEK-293 cells, known for their convenience in engineering and their use in biopharmaceutical manufacturing^7,41,47^, but it also works in a variety of mammalian cells (Extended Data Fig. 6).

### Stimulated insulin release in encapsulated HEK_EMPOWER_ cells

**Fig. 3:**
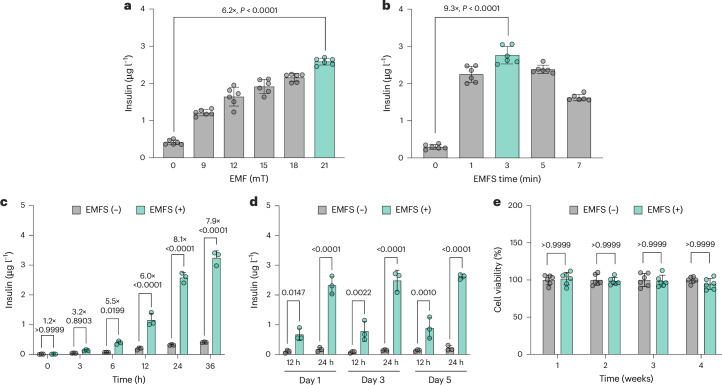
Electromagnetically stimulated gene expression in microencapsulated HEK_EMPOWER_ cells. **a**, Magnetic-field-dependent insulin expression (stimulation time, 3 min). **b**, Stimulation-time-dependent insulin expression (magnetic field, 21 mT, 1 kHz). **c**, Time-dependent insulin production during 36 h after an EMFS stimulation of 21 mT, 1 kHz, 3 min. Profiling was started immediately after EMF stimulation. **d**, Reversibility of insulin production. The cells were alternatively stimulated with an EMFS of 21 mT for 3 min (on) or unstimulated (off) at 24-h intervals. The cell culture medium was renewed each time the EMF stimulation was switched from on-to-off or from off-to-on. **e**, Viability of HEK_EMPOWER_ following daily 3-min EMF stimulation for 4 weeks (21 mT, 1 kHz, 3 min per day), compared with unstimulated HEK_EMPOWER_ cells. All data are presented as mean ± s.d.; *n* = 6 (**a**,**b**) and *n* = 3 (**c**,**d**) independent experiments. *P* values in **a**–**c** were calculated versus the corresponding non-stimulated control. The induction factors were calculated between non-stimulated (EMFS (−)) and stimulated (EMFS (+)) groups. Statistical significance was analysed by two-way ANOVA with Tukey’s test (**a**,**b**) and one-way ANOVA with Dunnett’s multiple comparisons test (**c**,**d**). Source data

**Extended Data Fig. 7:**
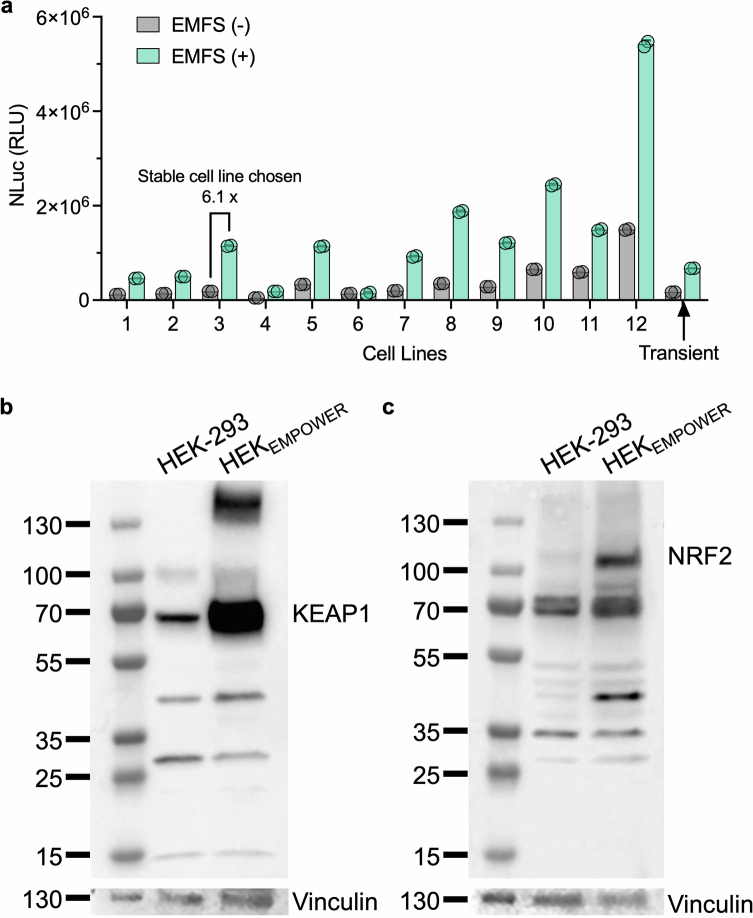
Screening of monoclonal HEK-293 cell lines based on NLuc expression in response to electromagnetic stimulation. (**a**) The EMPOWER system is based on the constitutive expression of KEAP1 (ITR-P_hCMV_-KEAP1-P2A-BlastR-pA-ITR, pJH1054) and NRF2 (ITR-P_hCMV_-NRF2-pA: P_RPBSA_-ECFP-P2A-PuroR-pA-ITR, pJH1101), and four-tandem ARE (DART4)-controlled NLuc reporter followed by mouse insulin (mINS) (ITR-P_DART4_-NLuc-P2A-mINS: P_mPGK_-ZeoR-pA-ITR, pJH1196). All the constructs are flanked by inverted terminal repeats (ITR) for SB100X-based Sleep Beauty transposase recognition. CBCFO concentration was fixed at 50 μg/10^6^ cells. Electromagnetic stimulation: 1 kHz, 21 mT, 3 min. All data are presented as means ± s.d.; n = 2 independent experiments. Western blot analysis of KEAP1 (**b**) and NRF2 (**c**) levels in parental (HEK-293) and HEK_EMPOWER_ cells. Vinculin was used as a loading control. Source data

**Extended Data Fig. 8:**
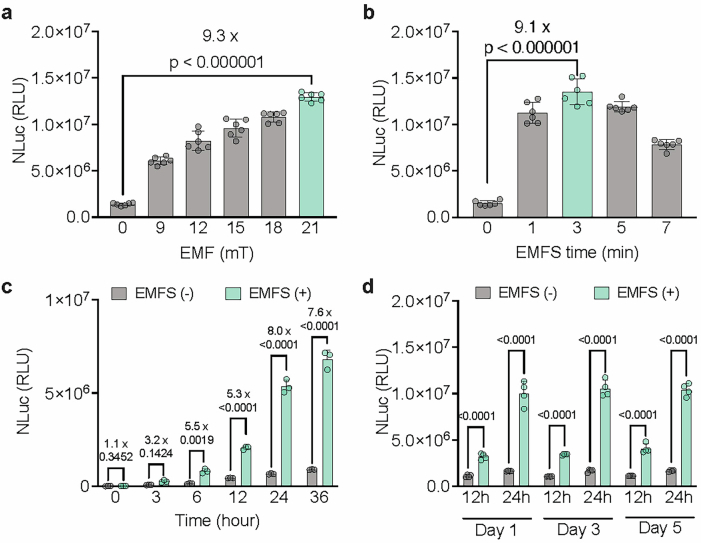
NLuc expression of encapsulated HEK_EMPOWER_ cells. (**a**) Field-strength-dependent NLuc expression (1 kHz, 3 min). (**b**) Stimulation-time-dependent NLuc expression (1 kHz, 21 mT). (**c**) Time-dependent expression over 36 h (1 kHz, 21 mT, 3 min). (**d**) Reversible expression (1 kHz, 21 mT, 3 min). The CBCFO concentration was fixed at 50 μg/10^6^ cells. P values and fold changes in (**a**, **b**, **c**) were calculated versus the corresponding non-stimulated control. All data are presented as means ± s.d.; (**a** and **b**) n = 6; (**c**) n = 3; (**d**) n = 4 independent experiments. Statistical significances were calculated via two-way ANOVA Dunnett’s multiple comparison tests. Source data

To construct the EMPOWER system for EMF-controlled insulin production and release in human cells, we first established stable HEK-293 cell lines engineered for constitutive expression of KEAP1 (*ITR-P*_*hCMV*_*-KEAP1-P2A-BlastR-pA-ITR*, pJH1054), NRF2 (*ITR-P*_*hCMV*_*-NRF2-pA:P*_*RPBSA*_*-ECFP-P2A-PuroR-pA-ITR*, pJH1101) and NRF2-dependent expression of insulin (*ITR-P*_*DART4*_*-NLuc-P2A-mINS-pA:P*_*mPGK*_*-ZeoR-pA-ITR*, pJH1196; P_DART4_, *O*_*ARE4*_*-P*_*hCMVmin*_). Nanoluciferase (NLuc) was used as a bioluminescent reporter for screening. The best-in-class monoclonal cell line, HEK_EMPOWER_, exhibited ectopic KEAP1 and NRF2 expression and showed the highest NLuc fold induction (Extended Data Fig. 7). To customize HEK_EMPOWER_ cells for implantation, they were mixed with CBCFO nanoparticles and enclosed in clinically licensed alginate microcapsules^48^ to shield the engineered cells from the host immune system while enabling diffusion of nutrients and the release of biopharmaceuticals. The performance of the HEK_EMPOWER_-containing implants was validated under a Helmholtz-coil-based uniform EMF. The magnetic field strength (Fig. 3a) and stimulation time dependence (Fig. 3b) of insulin production were evaluated. The highest insulin level of 2.76 ± 0.45 μg l^−1^ was obtained under an EMFS of 21 mT and 3 min, which is consistent with the results for NLuc expression (Extended Data Fig. 8). A kinetic study revealed that stimulated insulin production from the HEK_EMPOWER_ cells reached a significant level in the culture supernatant within 3 h and was maintained for over 24 h (Fig. 3c). We also confirmed the excellent reversibility in on/off stimulation patterns at 24-h intervals over 5 days (Fig. 3d and Extended Data Fig. 8d). Under standard stimulation conditions (1 kHz, 21 mT, 3 min), transgene expression compared favourably with reported levels of ROS-triggered gene expression^49^, and daily EMF exposure (21 mT, 1 kHz, 3 min per day) had no impact on cell viability during the experimental period of 4 weeks (Fig. 3e), suggesting that the EMPOWER system was operating at near-optimal performance (Figs. 2 and 3).

### Electromagnetically powered glucose homeostasis in T1D

**Fig. 4:**
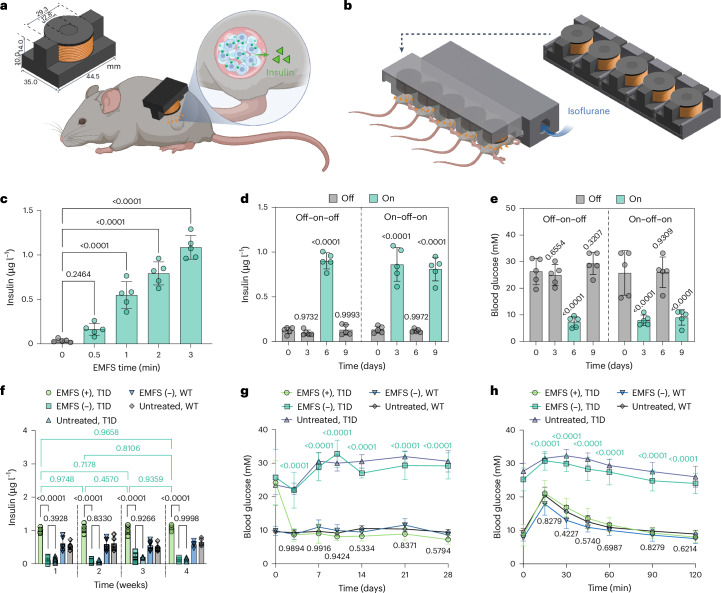
In vivo evaluation of HEK_EMPOWER_ cells for wireless-controlled treatment of T1D. **a**, Scheme illustrating the magnetic field stimulation of encapsulated HEK_EMPOWER_ cells implanted in the dorsoventral side of mice, using a centimetre-sized single-coil device. **b**, Scheme showing the simultaneous stimulation of an experimental group of mice with a parallel assembly of single-coil devices. **c**, Stimulation-time-dependent tunability of insulin secretion (21 mT, 1 kHz, 0–3 min). **d**,**e**, Reversibility of EMF-controlled insulin (**d**) and blood-glucose (**e**) levels. Microencapsulated subcutaneous HEK_EMPOWER_ implants were exposed to alternating on-to-off and off-to-on EMF stimulation every 3 days (on: 21 mT, 1 kHz, 3 min; off: unstimulated). **f**,**g**, Fasting blood-insulin (**f**) and blood-glucose (**g**) levels were recorded before implantation (week 0) and for up to 4 consecutive weeks after implantation of HEK_EMPOWER_ cells in T1D mice stimulated for 3 min (21 mT, 1 kHz): EMFS (+) group. T1D and WT mice groups with non-stimulated HEK_EMPOWER_ cell implants (EMFS (−)), and without implants (untreated) were used as controls. Over the entire treatment period of 4 weeks fed WT mice maintained average blood-insulin levels of 2.1 ± 0.8 µg l^−1^. **h**, Intraperitoneal GTT was performed on mice 3 days after implantation of microencapsulated cells and after fasting for 8 h. Data are presented as mean ± s.d., *n* = 5 (**c**–**g**) and *n* = 10 (**h**) biological replicates. *P* values in **d**,**e** were calculated between the indicated data and the initial (day 0) unstimulated T1D control. *P* values in **g**,**h** were calculated versus the corresponding non-stimulated control (black, bottom) and WT control (green, top). Statistical significance in **d**–**h** was analysed with two-way ANOVA Dunnett’s multiple comparison tests. Mouse schematic illustrations created with BioRender.com. Source data

For in vivo validation, we designed a single-coil EMF generator with an E-shaped iron core (Fig. 4a). The EMF from this single-coil device reached 20–22 mT at a plane 3–5 mm from the coil surface, which matches the depth of subcutaneous implantation in mice. This device generates a magnetic field gradient rather than the uniform field from the Helmholtz-coil device. The device was able to stimulate transgene expression from the encapsulated cells in vitro, and no significant difference was observed compared with the Helmholtz-coil device (Supplementary Fig. 7). For in vivo single-coil EMFS, five devices were fitted into a 3D-printed holder to facilitate parallel stimulation of mice (Fig. 4b and Supplementary Fig. 8).

**Extended Data Fig. 9:**
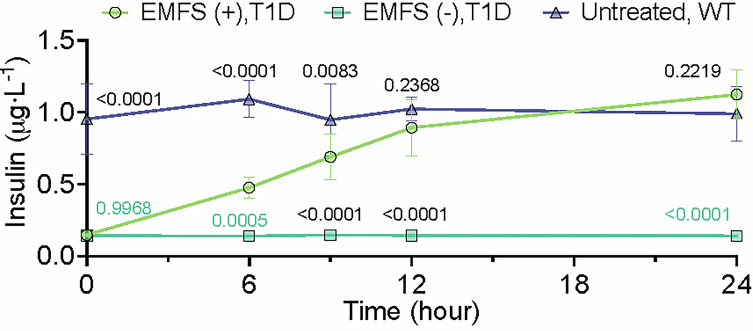
EMF-stimulated blood-insulin levels in type-1 diabetic mice. Type-1-diabetic mice implanted with microencapsulated HEK_EMPOWER_ cells were stimulated with EMF (21 mT, 1 kHz, 3 min). Blood insulin reached wild-type levels within 12 h. Data are presented as mean ± s.d., n = 5 biological replicates. Statistical significance of differences between EMF-stimulated and unstimulated control groups was analysed by two-way ANOVA with Dunnett’s multiple comparisons test. Source data

**Extended Data Fig. 10:**
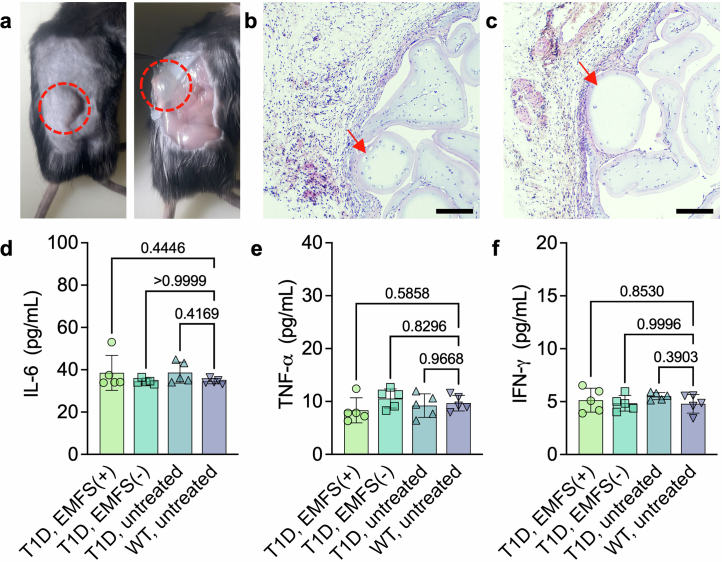
Impact of subcutaneous EMPOWER implants in mice. (**a**) Pictures of the implantation site of a representative T1D mouse subcutaneously implanted with the EMPOWER system and treated for four weeks with daily EMF stimulation (21 mT, 1 kHz, 3 min). The implant site is encircled and does not show any macroscopic signs of inflammation. (**b**, **c**) Histological analyses of tissue sections around the implant site of EMF-stimulated (**b**) and unstimulated (**c**) T1D mice. Microcapsules containing HEK_EMPOWER_ cells are indicated with arrows. Scale bar, 100 μm. (**d**-**f**) Profiling of key inflammatory cytokines, IL-6 (**d**), TNF-α (**e**) and IFN-γ (**f**), in the bloodstream of T1D mice four weeks after implantation of the EMPOWER system and daily EMF stimulation (21 mT, 1 kHz, 3 min). All data are presented as means ± s.d.; n = 5 biological replicates. Statistical significance was analysed by two-way ANOVA with Dunnett’s multiple comparisons test. Source data

The encapsulated HEK_EMPOWER_ cells implanted in T1D mice were subjected to a 3-min magnetic field stimulation (EMFS (+) T1D group) using the single-coil devices. Insulin secretion kinetics matched those found for other transcription-control modalities and the insulin levels were consistent with those in previous studies using experimental T1D as a proof-of-concept model (Extended Data Fig. 9)^7,12,41,47,50,51^. The insulin secretion levels of HEK_EMPOWER_ could be adjusted by varying the EMF stimulation time (Fig. 4c) and the glycaemic control was fully reversible; switching the EMFS from off-to-on or from on-to-off every 3 days resulted in corresponding changes in insulin (Fig. 4d) and blood-glucose levels (Fig. 4e). The EMFS-driven secretion of insulin (Fig. 4f) from the HEK_EMPOWER_ cells attenuated blood-glucose levels and subsequently maintained normoglycaemia in the T1D mice (Fig. 4g). Furthermore, EMFS-triggered insulin production by the HEK_EMPOWER_ cells ameliorated postprandial glycaemic excursions in glucose tolerance tests (GTTs) and restored normoglycaemic levels (Fig. 4h). Real-time glycaemic measurements confirmed that daily stimulation of the HEK_EMPOWER_ cells for 3 min could restore normoglycaemic levels in T1D mice and maintain glucose homeostasis for at least 4 weeks without any hypoglycaemic excursion. No significant difference in blood glucose or insulin levels was observed in non-stimulated wild-type (WT) mice implanted with HEK_EMPOWER_ cells (EMFS (−) WT group) compared with non-treated WT mice. This confirms non-leakiness of the EMPOWER system and is consistent with the absence of hypoglycaemic episodes. At the end of the treatment period, the animals showed no sign of macroscopic (Extended Data Fig. 10a) or systemic inflammation (Extended Data Fig. 10b–d), and histological analyses of the implantation site indicated that the EMPOWER capsules remained in place, intact and unaffected by EMF stimulation (Extended Data Fig. 10e,f). The body weight gain (1.5 ± 0.6 g per mouse), daily food intake (6.5 ± 0.8 g per mouse) and water consumption (7.7 ± 1.6 ml per mouse) of EMF-stimulated T1D mice were identical to those of WT mice in the terminal phase of the 4-week treatment period.

## Conclusions

EMFs represent promising, minimally invasive control modalities for next-generation gene- and cell-based therapies. First-in-class magnetic stimulation methodologies reported so far mostly use membrane channels or receptors conjugated to inorganic nanoparticles activated by thermal or mechanical coupling^8,15,31^. However, challenges still remain associated with receptor and channel functionalization and intracellular trafficking as well as off-target effects and toxicities, limited robustness, tunability and clinical translation of these methods^22,27,29,30^. Instead, our work utilizes modified multiferroic nanoparticles to communicate with cytoplasmic ROS sensors KEAP1/NRF2, affording a nanoparticle–cell interface to drive transgene expression via synthetic promoters for wireless electromagnetic cell therapy. To test this approach, we focused on T1D, one of the dynamically most challenging chronic diseases, requiring meticulous blood-glucose control and daily insulin administration. In a T1D mouse model, daily EMFS (21 mT, 1 kHz, 3 min.) of subcutaneously implanted, microencapsulated HEK_EMPOWER_ cells was sufficient to drive transgene expression of insulin at a level sufficient to produce sustained normoglycaemia. Our proof-of-concept study successfully restored normoglycaemia in a mouse model of experimental T1D throughout the 4-week experimental period, demonstrating dynamically robust, reversible and tunable in vivo control. The EMPOWER system compared favourably in performance with established cell-based therapeutic modalities using chemical^7,41,49,52^ and physical stimuli^12,13^ with identical cell-encapsulation technology, which has been validated for longevity^53^ and in human clinical trials^48^.

The CBCFO nanoparticles used here exhibit efficient coupling between magnetostrictive and piezoelectric composites^45^, while the bio-originated, positively charged polymer chitosan improves biocompatibility and cell adhesion^54^. In addition to shielding the bare ferric oxides from the cellular environment, chitosan also enables the short-lived ROS generated by the CBCFO nanoparticles to escape from the endosomes into the cytoplasm via the proton sponge effect^46^. Indeed, such multiferroic nanoparticles have been directly injected into the brain or blood circulation for deep neuron stimulation^26^, guided central nervous delivery^55^ and dissociation of Alzheimer’s β-amyloid aggregates^33^. A key advantage of our system is that cellular stimulation can be triggered at a much lower dose of nanoparticles (50 μg per 10^6^ cells, over 20 times lower than in the aforementioned applications)^56^. The alginate-microencapsulated implants also minimize the risk of liver damage^53^ associated with the direct administration of nanoparticles^27^. In addition, cellular ROS levels increased immediately after stimulation and then declined within 3–6 h, and the KEAP1/NRF2 system recognizes this ROS peak, not a gradual accumulation of ROS^14^, as typically observed in ROS-signalling systems^57^. Such kinetics limit the adverse effect of ROS on HEK_EMPOWER_ cells, as evidenced by the reversibility of the stimulation of therapeutic protein expression.

A low-frequency EMF of 1 kHz imposes a negligible magnetothermal effect or mechanical force on the cells^22^. More importantly, because even chemical ROS inducers producing systemic ROS surges have no apparent impact on cell physiology or metabolism^14^, EMF-triggered ROS induction confined to the vicinity of intracellular CBCFO nanoparticles should bear little risk of potential side effects. Additionally, our work highlights the use of weak EMFs (up to 21 mT), much weaker than those used in MRI scanners (in the tesla range), promising safety in clinical use. This level of EMFS can be achieved by a single induction coil with a fixed coil structure and input parameters (akin to wireless phone chargers), and tuned by adjusting a single parameter, stimulation time, avoiding the need for complex software or electronic implants. We believe that this kind of interface between programmable electronic devices and genetic therapies has the potential to dramatically streamline the treatment regimen for patients with chronic diseases.

## Methods

### Fabrication of CBCFO

CFO nanoparticles were synthesized according to the literature with modifications^33^. To prepare CFO nanoparticles, iron(III) chloride hexahydrate (0.995 g) and cobalt(II) chloride (0.239 g) were mixed in deionized water (35 ml) containing hexadecyltrimethylammonium bromide (2.041 g). Sodium hydroxide solution (6 M) was then added dropwise to the mixture under continuous stirring to achieve a final pH of 11.0. After ultrasound stimulation for 30 min, additional hydrothermal treatment was applied to the mixture at 180 °C for 24 h in a 50-ml Teflon-lined stainless-steel autoclave. The resulting black precipitates were washed with deionized water and ethanol several times after cooling to room temperature.

To synthesize BCFO magnetoelectric nanoparticles, a sol–gel treatment was applied to the as-prepared CFO nanoparticles^33^. Briefly, CFO nanoparticles (50 mg) were dispersed into 30 ml ethylene glycol (catalogue number 324588, Sigma-Aldrich) containing bismuth(III) nitrate pentahydrate (0.160 g) and iron(III) nitrate nonahydrate (0.121 g). After 2 h of sonication, the sol mixture was moved to a vacuum oven and dried for 24 h. Next, the resulting gel-state mixture was preheated at 400 °C for 30 min to eliminate organic compounds and successively calcined at 500 °C for 90 min. The resulting BCFO nanoparticles were washed several times with deionized water and ethanol on a nylon membrane and collected with a neodymium permanent magnet after ultrasound treatment.

Chitosan (catalogue number 448877-50 G, Sigma-Aldrich) was first dissolved in 0.1-M NaCl to form a 0.1% solution after acidification with 1% acetic acid. Rhodamine B isothiocyanate (RITC)-labelled chitosan was prepared by dissolving RITC (40 µM, catalogue number CAY20653-100 mg, Cayman) in methanol and mixing it 1:1 with a 10 mg ml^−1^ chitosan solution under nitrogen protection, followed by dialysis against 0.1-M NaCl. The prepared BCFO nanoparticles were then dispersed and mixed in the chitosan solution (5 mg ml^−1^) by sonication for 1 h. The CBCFO nanoparticles were collected by centrifugation and washed with water three times. RITC-CBCFO nanoparticles were fabricated by mixing BCFO nanoparticles with RITC-labelled chitosan.

For cellular uptake, all nanoparticles were sonicated at 35 kHz for 30 min (Bandelin Electronic, RK100H) and filtered through a 0.22-µm filter (catalogue number P668.1, Carl Roth).

### Characterization of CBCFO

The morphology of the obtained CFO, BCFO and CBCFO nanoparticles was examined by TEM (FEI F30) and STEM (JEM-F200). The distribution of elements along the nanoparticles was studied by STEM EDX mapping (JEM-F200). The crystallographic structure of the nanostructures was analysed by XRD on a Bruker AXS D8 Advance 1 X-ray diffractometer, equipped with a copper target at a wavelength of 1.542 Å. The magnetic properties were evaluated by scanning probe microscopy (Bruker Dimension ICON) according to the magnetic force model. The zeta potential and the hydrodynamic size of samples were measured by a dynamic light scattering Zetasizer (Malvern, ZEN3600) in DPBS (0.01 M, pH 7.4). Relative charge separation and ROS induction from nanoparticles were evaluated by TA assay (3 mM, *λ*_ex_/*λ*_em_ = 310/430 nm) and MB assay (5 mM, *λ*_abs_ = 664 nm), respectively, using a plate reader (Tecan, Spark Reader). For TA and MB assays, an aqueous solution (400 μl) containing different nanoparticles was exposed to a magnetic field under constant agitation, and 100-μl aliquots of the supernatant were transferred to 96-well plates for colorimetric or fluorometric measurement.

### Magnetic field stimulation

Electromagnet-containing 3D-printed holders (Supplementary Figs. 5a and 8c) were designed to minimize the thermal effect on biological systems. Samples were exposed to a uniform EMF by placing them in the central area (5.8 cm × 5.8 cm) of a Helmholtz-coil-based device. The circuits (Supplementary Fig. 5c) for magnetic field stimulation were powered by custom-designed electrical drivers. The field strength generated by the Helmholtz-coil device was 9–21 mT and that generated by the single-coil device was 20–22 mT at a plane of 0.3–0.5 cm from the coil, with the frequency fixed at 1 kHz (sinusoidal). The amplitude of the applied alternating magnetic field was confirmed by a gaussmeter.

### Cell culture and engineering

#### Cell culture

Human embryonic kidney cells (HEK-293, ATCC, CRL-11268), human telomerase-immortalized mesenchymal stem cells (hMSC-TERT, RRID: CVCL_Z015), human liver cancer cell line (HepG2, ATCC, CRL-11997), Chinese hamster ovary cells (CHO-K1, ATCC, CCL-61), baby hamster kidney cells (BHK-21, ATCC, CCL-10) and mouse pituitary tumour cells (AtT-20, ATCC, CCL-89), were cultivated in Dulbecco’s modified Eagle’s medium (DMEM, catalogue number 52100-39, Thermo Fisher Scientific) supplemented with 100 mM proline (CHO-K1 only), 10% fetal bovine serum (FBS, catalogue number F7524, Sigma-Aldrich) and 1% (v/v) streptomycin/penicillin (catalogue number L0022, Biowest) at 37 °C in a humidified atmosphere containing 5% CO_2_.

#### Cell transfection

For transfection, 10^4^ cells (CellDrop BF Brightfield Cell Counter, DeNovix) were seeded per well in a 96-well plate (catalogue number 3599, Corning Life Sciences) 24 h before transfection by addition of 20 µl of a mixture containing 0.3 µg polyethyleneimine (PEI MAX, mol. wt 40,000, 1 μg μl^−1^ in double-distilled H_2_O, catalogue number 24765-2, Polysciences) and 0.1 µg plasmid DNA (equimolar concentrations for plasmid mixtures) per well. After 8 h, the mixture was replaced with a standard cultivation medium or nanoparticle medium suspension (100 µl) for further characterization.

#### Monoclonal cell line construction

HEK-293 cells (1.5 × 10^5^) were cotransfected with pJH1101 (*ITR-P*_*hCMV*_*-NRF2-pA: P*_*RPBSA*_*-ECFP-P2A-PuroR-pA-ITR*) (200 ng), pJH1054 (*ITR-P*_*hCMV*_*-KEAP1-P2A-BlastR-pA-ITR*) (550 ng), pJH1096 (*ITR-P*_*ARE*_*-NLuc-P2A-mINS:P*_*mPGK*_*-ZeoR-pA-ITR*) (400 ng) and pJH42 (*P*_*hCMV*_*-SB100X-pA*) encoding constitutive expression of a hyperactive Sleeping Beauty (SB) transposase (200 ng)^58^. After selection for two passages in culture medium supplemented with 2.5 μg ml^−1^ puromycin, 300 μg ml^−1^ blasticidin and 300 μg ml^−1^ zeocin, the resistant polyclonal population was divided by ECFP-based FACS-mediated single-cell sorting into 48 monoclonal cell lines. Twelve monoclonal cell lines with the highest ECFP-based fluorescence intensity were loaded with CBCFO nanoparticles (50 μg per 10^6^ cells) and stimulated by EMF (1 kHz, 21 mT, 3 min). HEK_EMPOWER_ (clone number 3), showing best-in-class EMF-stimulated transgene-fold induction, was chosen for further studies (Extended Data Fig. 7a).

### Microencapsulation and implantation of HEK_EMPOWER_ cells

To protect HEK_EMPOWER_ cells from the mouse immune system while permitting the exchange of nutrients and release of therapeutic proteins, we used a clinical trial-validated alginate-based encapsulation technology^48^. HEK_EMPOWER_ cells were encapsulated in alginate/poly(l-lysine)/alginate microcapsules with a diameter of 400 µm by treating a mixture of 9.0 × 10^7^ cells with 18 ml alginate (w/v, 1.6%; Na-alginate, catalogue number 71238, Sigma-Aldrich) in an encapsulator (Inotech Encapsulator IE-50R, EncapBiosystems) equipped with a 200-μm nozzle. A 20-ml syringe was operated at a flow rate of 20 ml min^−1^ with a vibration frequency of 1.2 kHz and 1.2 kV voltage for bead dispersion. A 100-ml poly(l-lysine) 2000 (w/v, 0.05%; catalogue number 25988-63-0, Alamanda Polymers) solution and a 100-ml 0.03% alginate solution were sequentially used to form the microcapsules. For delivery, 2.5 × 10^6^ encapsulated cells in 0.5 ml serum-free DMEM were subcutaneously implanted through a 3-ml syringe (catalogue number 9400038, Becton Dickinson) with a 0.7-mm × 30-mm needle (catalogue number 30382903009009, Becton Dickinson).

### Animal experiments

#### Preparation of experimental mouse models

C57BL/6JRJ mice were kept and monitored in groups (*n* = 5) in an environment controlled at 21 ± 2 °C and 55 ± 10% humidity and maintained under a 12-h reverse light–dark cycle, with free access to standard diet and water. All procedures were performed in compliance with Swiss animal welfare regulations, approved by the Veterinary Office of the Canton Basel-Stadt, Switzerland (license number 2996_34477), the French Republic (project number DR2018-40v5 and APAFIS number 16753) and the People’s Republic of China (Institutional Animal Care and Use Committee of Westlake University, protocol ID20-009-XMQ). The experiments were conducted by P.G.R. (license number LTK 5507), G. Charpin-El Hamri (number 69266309; University of Lyon, Institut Universitaire de Technologie) or by S. Xue (Westlake University). Two groups of mice were utilized: WT and experimentally induced T1D mice. To induce the T1D condition, male WT mice (8–9 weeks old, 18–23 g) were intraperitoneally injected with streptozotocin (STZ; 75 mg kg^−1^, 0.2 M citrate buffer, pH 4.2; Sigma-Aldrich, catalogue number S0130) for 4 consecutive days following a 6-h fasting period^59^. Control WT mice from Janvier Labs (18–23 g) received identical injections without STZ. At 10 days after the final injection of STZ, fasting blood-glucose levels were measured using ContourNext test strips and a ContourNext ONE reader (Ascensia Diabetes Care; catalogue numbers 84191451 and 85659367) to confirm persistent hyperglycaemia and T1D status in the STZ-treated group.

#### Experimental procedure

Microencapsulated HEK_EMPOWER_ cells with CBCFO nanoparticles (50 μg per 10^6^ cells) were subcutaneously implanted in the experimental and control groups. The hair on the dorsoventral side of the mice was completely shaved, and the animals were anaesthetized with 4% isoflurane and maintained under 2% isoflurane during surgery. Microencapsulated HEK_EMPOWER_ cells were injected subcutaneously (0.5 ml DMEM, 5 × 10^6^ cells) on the dorsoventral side using a 5-ml syringe with a 21-gauge needle to reduce the risk of aseptic loosening. After a 24-h stabilization period, the HEK_EMPOWER_ cells were wirelessly stimulated using a portable (single-coil-based) device (Fig. 4b) for 3 min once every 24 h in the EMFS (+) group. For the rest of each day, treated animals were not restrained. The single-coil devices (*n* = 5) were fitted into a 3D-printed holder (Supplementary Fig. 8d) and a rectangular tunnel (with five parallel holes, Supplementary Fig. 8b) was used to maximize efficiency and facilitate parallel experiments. The animals were fasted for 6 h before measuring blood-glucose and insulin levels. For the GTT experiment, treated animals were intraperitoneally injected with 1.5 g kg^−1^ glucose and glycaemia was recorded at regular intervals over 2 h. Real-time blood-glucose monitoring was performed at regular time points over a period of 4 weeks after a fasting period of 6 h. Alongside glycaemic levels, the corresponding blood insulin levels were also measured and compared with those of untreated WT and T1D groups.

#### Blood collection

The level of blood glucose was monitored periodically using ContourNext test strips and a ContourNext ONE reader (catalogue numbers 84191451 and 85659367, Ascensia Diabetes Care)^60^. Blood insulin levels were assessed in serum samples collected in Microtainer serum separator tubes (centrifuged at 6,000*g* for 10 min at 4 °C; catalogue number 365967, Becton Dickinson) with an ultrasensitive ELISA assay (catalogue number 10-1247-01, Mercordia).

#### Histology

Microencapsulated HEK_EMPOWER_ and surrounding tissue were explanted from EMF-stimulated and unstimulated mice and fixed overnight in 10% buffered formalin (100 ml 40% formalin, 900 ml double-distilled H_2_O, 4 g l^−1^ NaH_2_PO_4_, 6.5 g l^−1^ Na_2_HPO_4_, pH 7). The tissue samples were trimmed, dehydrated in increasing concentrations of ethanol, cleared with xylene, embedded in paraffin wax, processed into 5-µm slices using an EXAKT 300 CP system (EXAKT Technologies) and stained with haematoxylin and eosin. The tissue sections were analysed by light microscopy (Olympus CKX53) and images were acquired with an Olympus DP75 camera.

### Statistics and reproducibility

The data presentation, sample size of biological replicates (*n*), statistical analysis and significance of differences are shown in the figure legends. All in vitro experiments were repeated at least twice unless otherwise stated. For the mouse experiments, biological replicates (*n* = 5 mice per group) were randomly assigned to different experimental groups. The details are described in each figure legend. To determine the statistical significance of differences in the case of multiple comparisons we used GraphPad Prism 10 (v.10.1.0, GraphPad Software) and a two-tailed, unpaired, Student’s *t*-test and one-way or two-way analysis of variance (ANOVA). No statistical methods were used to prespecify sample sizes, but our sample sizes are the same as previously reported^12,14^. Data distribution was assumed to be normal, but was not formally tested. All investigators involved in this study were blinded to group allocation during data collection and analysis. No animals or data points were excluded from the analyses for any reason.

### Reporting summary

Further information on research design is available in the Nature Portfolio Reporting Summary linked to this article.

## Online content

Any methods, additional references, Nature Portfolio reporting summaries, source data, extended data, supplementary information, acknowledgements, peer review information; details of author contributions and competing interests; and statements of data and code availability are available at 10.1038/s41565-025-01929-w.

## Supplementary information

Supplementary Information Supplementary Figures 1–8 and Supplementary Table 1

Reporting Summary

Supplementary Data Statistic source data for Supplementary Figures.

## Source data

Source Data Fig. 1 Statistical source data for Fig. 1.

Source Data Fig. 2 Statistical source data for Fig. 2.

Source Data Fig. 3 Statistical source data for Fig. 3.

Source Data Fig. 4 Statistical source data for Fig. 4.

Source Data Extended Data Fig. 1 Statistical source data for Extended Data Fig. 1.

Source Data Extended Data Fig. 1d Unprocessed western blots for Extended Data Fig. 1d

Source Data Extended Data Fig. 2 Statistical source data for Extended Data Fig. 2.

Source Data Extended Data Fig. 3 Statistical source data for Extended Data Fig. 3.

Source Data Extended Data Fig. 4 Statistical source data for Extended Data Fig. 4.

Source Data Extended Data Fig. 5 Statistical source data for Extended Data Fig. 5.

Source Data Extended Data Fig. 6 Statistical source data for Extended Data Fig. 6.

Source Data Extended Data Fig. 7 Statistical source data for Extended Data Fig. 7.

Source Data Extended Data Fig. 7 Unprocessed western blots for Extended Data Fig. 7b

Source Data Extended Data Fig. 8 Statistical source data for Extended Data Fig. 8.

Source Data Extended Data Fig. 9 Statistical source data for Extended Data Fig. 9.

Source Data Extended Data Fig. 10 Statistical source data for Extended Data Fig. 10.

## Data Availability

All data supporting the findings of this study are presented in the paper and the Supplementary Information. Source data are provided with this paper.
