# Supplementary material for: Electromagnetic wireless remote control of mammalian transgene expression
Source: Nat Nanotechnol. 2025 May 5;20(8):1071–8. doi: 10.1038/s41565-025-01929-w (PMC12373504; doi:10.1038/s41565-025-01929-w)
Supplement: Supplementary file 1 — Supplementary Figures 1–8 and Supplementary Table 1 [file 41565_2025_1929_MOESM1_ESM.pdf]

nature nanotechnology

Supplementary information https://doi.org/10.1038/s41565-025-01929-w

Electromagnetic wireless remote control of mammalian transgene expression

In the format provided by the authors and unedited

Table of contents

Supplementary Methods 2

Supplementary Figures 1-9 5

Supplementary Table 1 14

References 16

Supplementary Methods

Analytical assays

SEAP quantification. SEAP levels were profiled in cell culture supernatants using a colorimetric assay. A total of 100 µl SEAP assay 2× buffer (20 mM homoarginine, 1 mM $MgCl_2$ and 21% diethanolamine, pH 9.8) was mixed with 80 µl heat-inactivated (30 min at 65 °C) culture supernatant. After the addition of 20 µl substrate (120 mM p-nitrophenyl phosphate; Cat. No. AC128860100, Thermo Fisher Scientific), the absorbance was recorded for 30 min at 405 nm and 37 °C (Tecan Infinite 200 PRO) and SEAP levels were determined as described previously[1].

Endocytosis assay. Standard endocytosis profiling[2,3] included addition of endocytosis inhibitors nystatin (30 µg·mL^{-1}, N9150-20ML, Sigma-Aldrich) and chlorpromazine (20 µg·mL^{-1}, C-904-1ML, Supelco) to the cells 30 min before the addition of CBCFO nanoparticles followed by EMF stimulation (21 mT, 1 kHz, 3 min.) and profiling of SEAP expression.

NLuc quantification. The concentration of NLuc in cell culture supernatants and flood serum was measured by using the Nano-Glo Luciferase Assay System (Cat. No. N1110, Promega, Madison, WI, USA). In brief, 7.5 µl of each supernatant sample was mixed with 7.5 µl of Nano-Glo substrate-containing buffer (in a ratio of 1:50) in black 384-well plates (Cat. No. 781900, Greiner, Germany) and incubated at room temperature for 10 min. Total luminescence was measured with a Tecan Spark plate reader (Tecan Group AG, Maennedorf, Switzerland). Reversibility was profiled by cultivating microencapsulated $HEK_{EMPOWER}$ while switching EMF stimulation (21 mT, 1 kHz, 3 min.) ON or OFF, changing the cell culture media and profiling transgene expression at 24 h intervals.

MTT assay. To assess the percentage of viable cells with active metabolism, cells were seeded at 10^4/well in 96-well plates and treated with nanoparticles or/and exposed to a magnetic field. 10 µL MTT ((3-[4,5-dimethylthiazol-2-yl]-2,5-diphenyltetrazolium bromide) (Cat. No. 475989, Sigma-Aldrich) at 5 mg mL^{-1} in phosphate-buffered saline) was added 48 hours after treatment and culture was continued for 4 hours. After discarding the cell supernatant, 150 µl dimethyl sulfoxide (Cat. No. D8418, Sigma-Aldrich) was added to each well. After shaking, optical density (OD) was measured at 490 nm with 670 nm as a reference. To calculate the relative cell viability, the OD of non-treated cells was set to 100%.

ROS quantification. Cellular ROS were quantified with a Fluorometric Intracellular Ros Kit (Cat. No. MAK145, Merck). HEK-293 cells were incubated with nanoparticles for 24 h, then washed with 100 µL phosphate-buffered saline (PBS, Cat. No. 14190-094, Thermo Fisher Scientific), and the ROS assay agent was added (Cat. No. MAK145, Merck). Then, 100 µL stain solution obtained by mixing ROS

Detection Reagent Stock Solution and Assay Buffer (1:5000, v/v) was added for one hour and the cells were EMF-stimulated. The ROS levels were quantified by fluorescence assay normalised to blank assay fluorescence (520/605 nm, Tecan Spark Reader).

Extrusion of CBCFO nanoparticles from $HEK_{EMPOWER}$ cells. Native and microencapsulated $HEK_{EMPOWER}$ cells were cultivated in inserts (200 µL DMEM) placed in the bottom wells (1.2 mL DMEM, renewed daily) of 12-well insert plates (PET membrane, 3.0 µm, Cat. No. 210518324, VWR) and extruded CBCFO nanoparticles were profiled in 20 µL samples from the bottom wells.

Mitochondrial membrane potential (MMP) assay. To profile changes in mitochondrial membrane potential, cells were seeded in 96-well plates at a density of 8×10^3 per well, loaded with nanoparticles and optionally exposed to EMF. MMP was quantified using the JC-10-based kit according to the manufacturer's instructions (Cat. No. MAK159-1KT, Sigma-Aldrich).

Cytochrome C release assay. Cytosolic and mitochondrial cytochrome C was extracted from cells using the cytochrome C Release Assay Kit (Cat. No. ab65311, Abcam) and quantified by Western blotting (see below) using horseradish peroxidase-linked anti-mouse IgG (Cat. no. GENA931V-1ML, Sigma, 1:5,000 dilution).

Insulin quantification. Mouse insulin enzyme-linked immunosorbent assay (ELISA) kits were used to quantify recombinant mouse insulin levels in culture supernatants (Cat. No. 10-1247-01, Mercodia, Uppsala, Sweden) and mouse serum (Cat. No. 10-1249-01, Mercodia), according to the manufacturer's instructions.

Inflammatory cytokine assay. Systemic inflammatory blood cytokine levels were quantified in mouse serum using ELISA kits (IL-6, Cat. No. ab100712; IFN-γ, Cat. No. ab282874; TNF-α, Cat. No. ab208348, all from Abcam).

Energy density calculation

EMF energy density providing optimal transgene expression (B_{max} = 21 mT, f = 1 kHz, 3 minutes) was calculated as follows:

$$B(t) = B_{max}\sin(2\pi ft) \quad (1)$$

$$u(t) = B(t)^2/(2\mu_0) \quad (2)$$

$$E_{mag} = \int u(t)dt \quad (3)$$

where B(t) is the magnetic field intensity, u(t) is the energy density, E_{mag} is the total time integration of the EMF energy density; B_{max} = 21 mT, f = 1000 Hz, $\mu_0 = 4\pi \times 10^{-7}$ $H \cdot m^{-1}$, t_{total} = 180 s. For three minutes, E_{mag} reaches 1.5×10^4 $J \cdot s \cdot m^{-3}$.

Flow cytometry and fluorescence microscopy

To evaluate the endosome escape capacity of nanoparticles, HEK-293 cells were seeded in 24-well plates (1×10^5 cells per well), incubated with different nanoparticles, and sequentially stained with Lyso-Tracker Green (50 nM, Cat. No. 8783, Cell Signaling) at 37 °C for 1 h, and Hoechst-33342 ($mg \cdot mL^{-1}$, Cat. No. R376055, Invitrogen) at room temperature for 20 min. These counterstained cells were subjected to microscopic and flow cytometric analysis. For fluorescence microscopy, cells stained in the same way were kept under 5% CO_2 at 37 °C and imaged under a cooled CCD camera. The images were analyzed, and Pearson's correlation coefficients were calculated by FIJI ImageJ with the Colocalization plugin.

Western blotting

5 x 10^6 exponentially growing cells were lysed in ice-cold radioimmunoprecipitation assay buffer (150 mM NaCl, 50 mM Tris-HCl 8.0, 1% Nonidet P-40 (NP40), 0.5% sodium deoxycholate, 0.1% SDS, 1 mM sodium orthovanadate, 1 mM NaF and protease inhibitors; all from Roche) during 30 min at 4°C with continuous agitation, followed by centrifugation at 15,000 x g for 20 min at 4°C. The supernatants were harvested and incubated on ice. The total protein concentration was quantified with a Pierce BCA Protein Assay kit (Cat. No. 23225, Thermo Fisher). Then, the samples were boiled in 2x Laemmli buffer (BioRad, Cat. No. 1610737) at 95 °C for 5 min. Subsequently, 10 µg of the sample was resolved by SDS–PAGE and transferred to a polyvinylidene fluoride membrane (Cat. No. 88518, Thermo Fisher) in transfer buffer (25 mM Tris, 190 mM glycine and 20% methanol). After the transfer, membranes were blocked with 3 % non-fat milk at 4°C and incubated with KEAP1 (Cat. No. ab227828, Abcam; 1:5,000 dilution) and NRF2 (Cat. No. ab137550, Abcam, 1:5,000 dilution) primary antibodies overnight at 4°C, followed by incubation with secondary antibody (anti-rabbit horseradish peroxidase-linked IgG; Cat. No. GENA934-1ML, Sigma-Aldrich, 1:5,000 dilution). The proteins were visualized after adding the chemiluminescence substrate (Pierce ECL Western Blotting Substrate, Cat. No. 32106, Thermo Fisher) using a chemiluminescence detection system (FusionPulse TS, Cat. No. 121172301, v.5.12a). Rabbit vinculin (Cat. No. 4650, Cell Signaling Technology, 1:2,000 dilution) was used as a loading control.

a

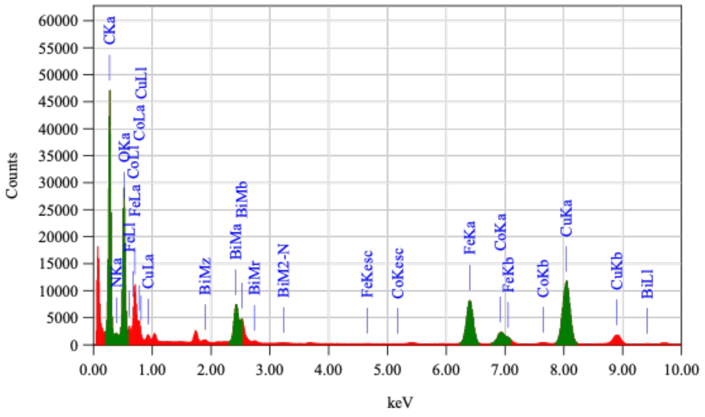


```
Acquisition Parameter
Instrument    : JEM-F200(HRP)
Acc. Voltage : 200.0 kV
Probe Current: 7.47500 nA
PHA mode      : T4
Real Time     : 901.09 sec
Live Time     : 837.26 sec
Dead Time     : 6 %
Counting Rate: 1822 cps
Energy Range :  0 - 40 keV
```

Thin Film Standardless Standardless Quantitative Analysis
Fitting Coefficient : 0.1185

Element	(keV)	Mass%	Counts	Sigma	Atom%	Compound	Mass%	Cation	K
C K* (Excluded)									
N K	0.392	0.83	3270.12	0.03	2.33				1.3486
O K (Ref.)	0.525	27.61	147438.53	0.13	68.16				1.0000
Fe K	6.398	21.04	110085.97	0.14	14.88				1.0208
Co K !	6.924	10.56	28429.52	0.14	7.08				1.9834
Cu K* (Excluded)									
Bi M	2.419	39.96	77309.80	0.27	7.55				2.7606
Total		100.00			100.00				

b

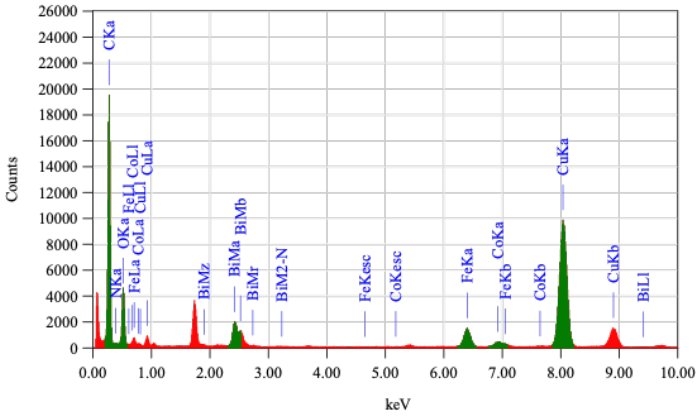


```
Acquisition Parameter
Instrument    : JEM-F200(HRP)
Acc. Voltage : 200.0 kV
Probe Current: 7.47500 nA
PHA mode      : T4
Real Time     : 866.57 sec
Live Time     : 837.23 sec
Dead Time     : 3 %
Counting Rate: 715 cps
Energy Range :  0 - 40 keV
```

Thin Film Standardless Standardless Quantitative Analysis
Fitting Coefficient : 0.0960

Element	(keV)	Mass%	Counts	Sigma	Atom%	Compound	Mass%	Cation	K
C K* (Excluded)									
N K	0.392	1.21	995.32	0.07	2.05				1.3486
O K (Ref.)	0.525	21.64	23931.42	0.26	61.17				1.0000
Fe K	6.398	17.20	18636.91	0.27	18.39				1.0208
Co K !	6.924	8.78	4897.21	0.28	8.96				1.9834
Cu K* (Excluded)									
Bi M	2.419	51.16	20496.29	0.68	9.43				2.7606
Total		100.00			100.00				

Supplementary Figure 1. Energy-dispersive X-ray (EDX) spectra of nanoparticles. (a) CBCFO and (b) BCFO nanoparticles. Atom percentages were calculated after exclusion of C and Cu, which originate from the copper grid and the supporting carbon layer. The atom ratio of Co and Bi was similar in both nanoparticles, while in CBCFO, the percentage of N is increased due to the chitosan coating.

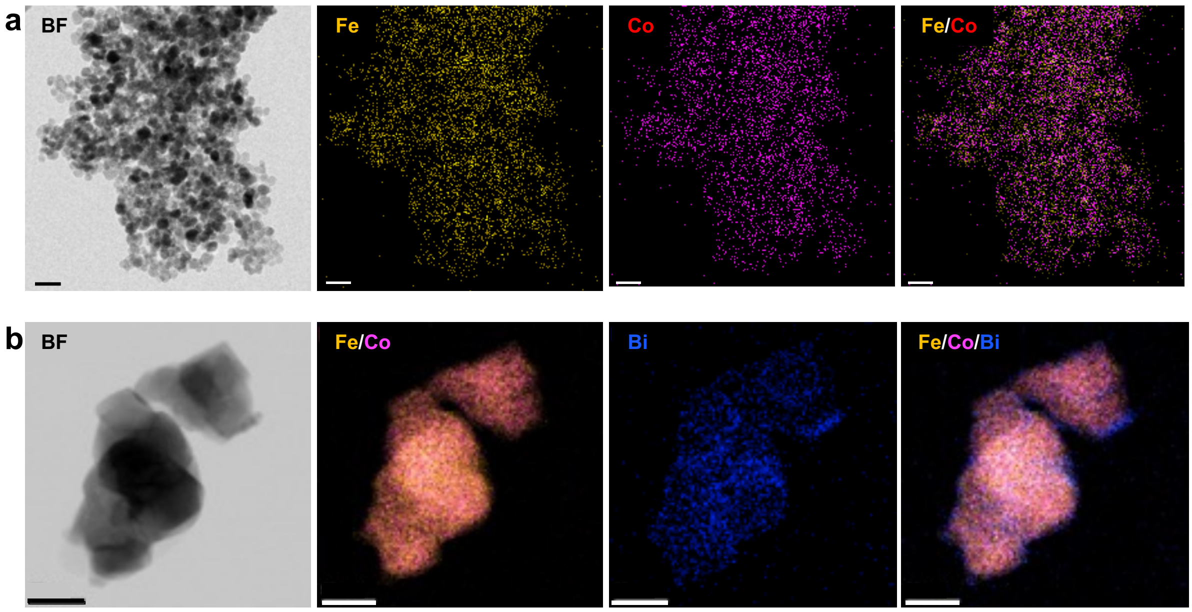

Supplementary Figure 2. STEM bright-field (BF) images and EDX mappings of CFO and BCFO nanoparticles. (a) BF image, EDX mappings of Fe, Co and overlay image (Fe/Co) of CFO. **(b)** BF image, EDX mappings of Fe, Co, Bi, and overlay images (Fe/Co) and (Fe/Co/Bi) of BCFO. The distribution of Bi and its colocation with Co/Fe elements are consistent with the core-shell structure of BCFO. Scale bar, 50 nm.

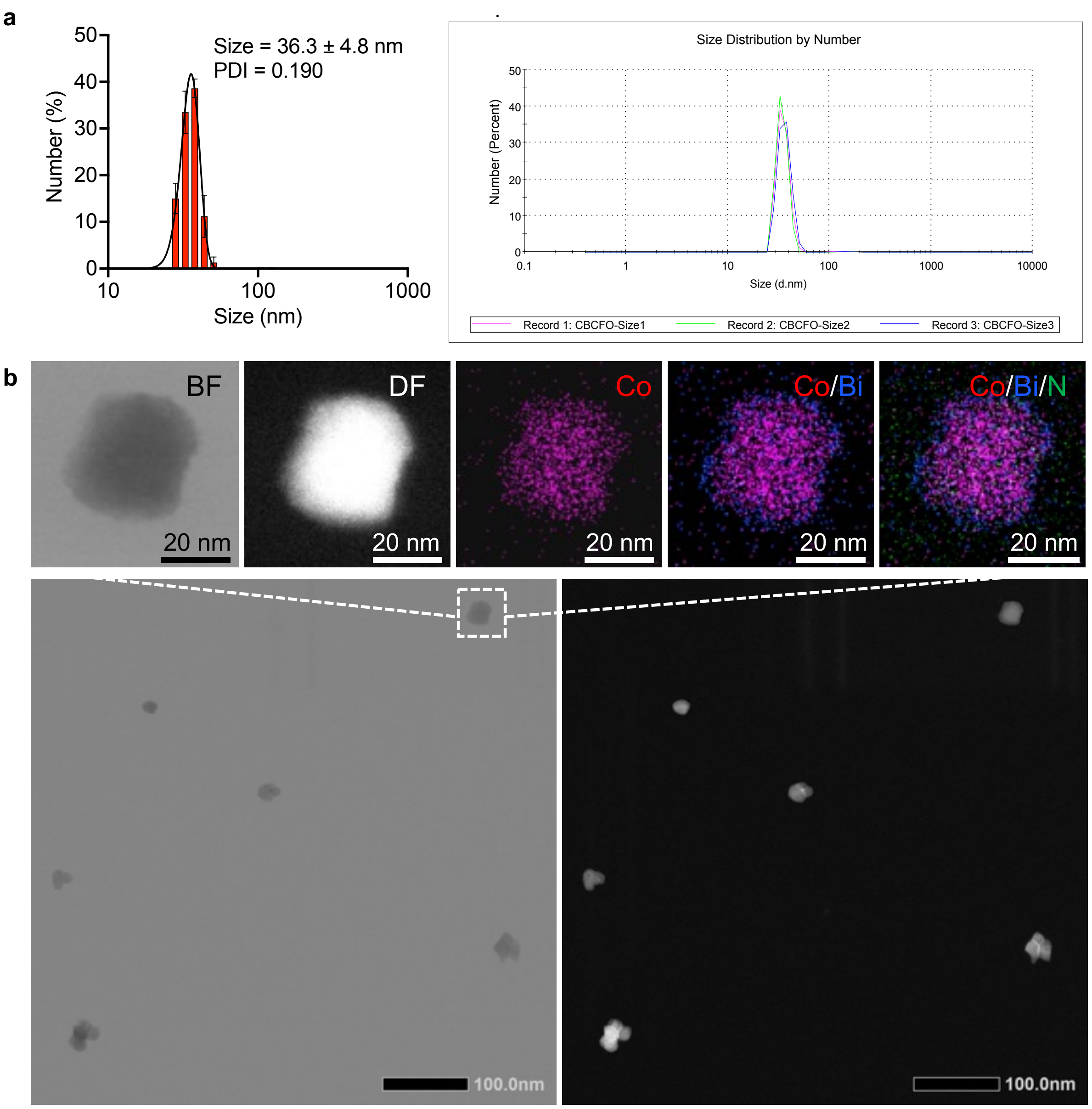

Supplementary Figure 3. Size distribution and polydispersity of CBCFO nanoparticles. **(a)** Dynamic laser scattering (DLS) of CBCFO. The hydrodynamic size distribution of CBCFO nanoparticles and polydispersity were averaged from five independent reads and replicated three times. (**b**) Bright-field (BF) and dark-field (DF) TEM images and EDX mapping of individual CBCFO nanoparticles.

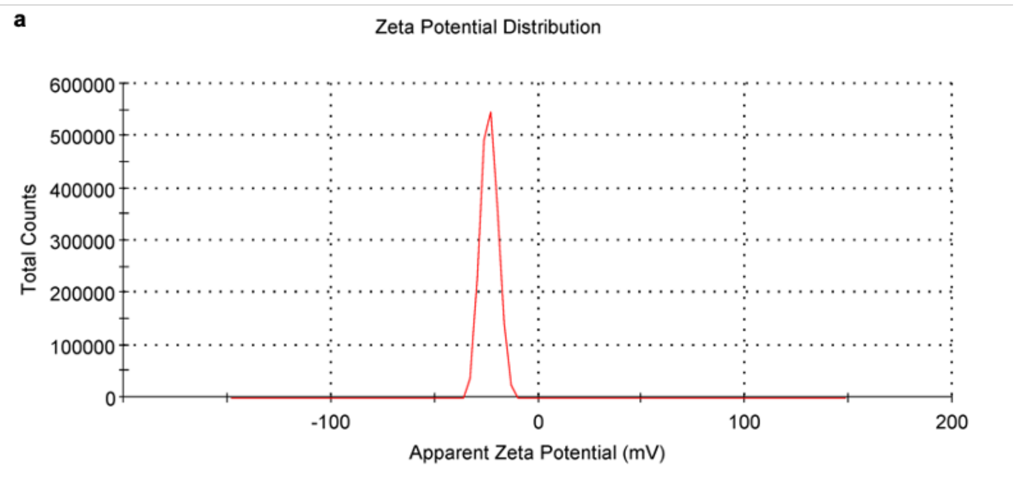

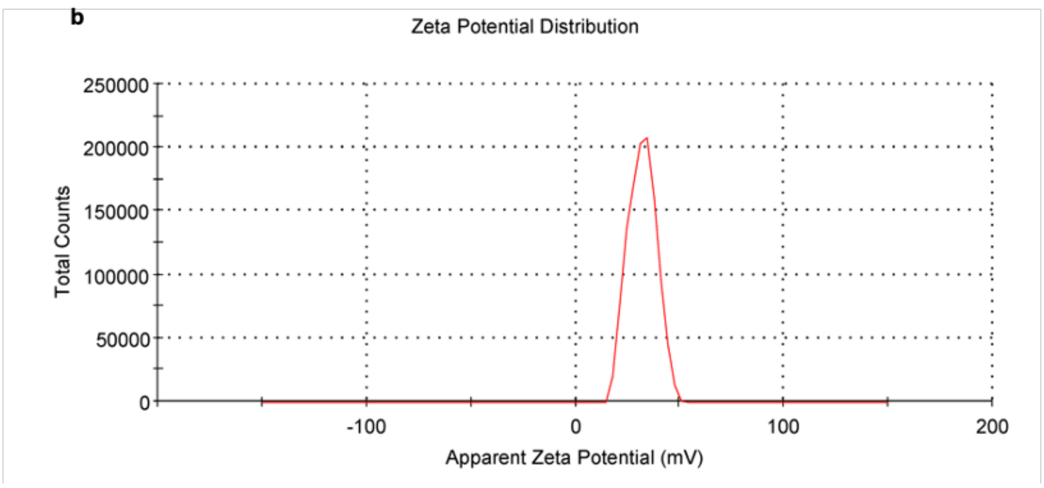

Supplementary Figure 4. Zeta potential distribution of nanoparticles. (a) Zeta potential of BCFO is negative (-22.5 ± 5.5 mV, mean ± SD) due to the metal-hydroxyl moieties in water. **(b)** Zeta potential of CBCFO nanoparticles is positive (31.6 ± 4.6 mV, mean ± SD) due to the surface modification of the amino-rich chitosan. All measurements were replicated 3 times.

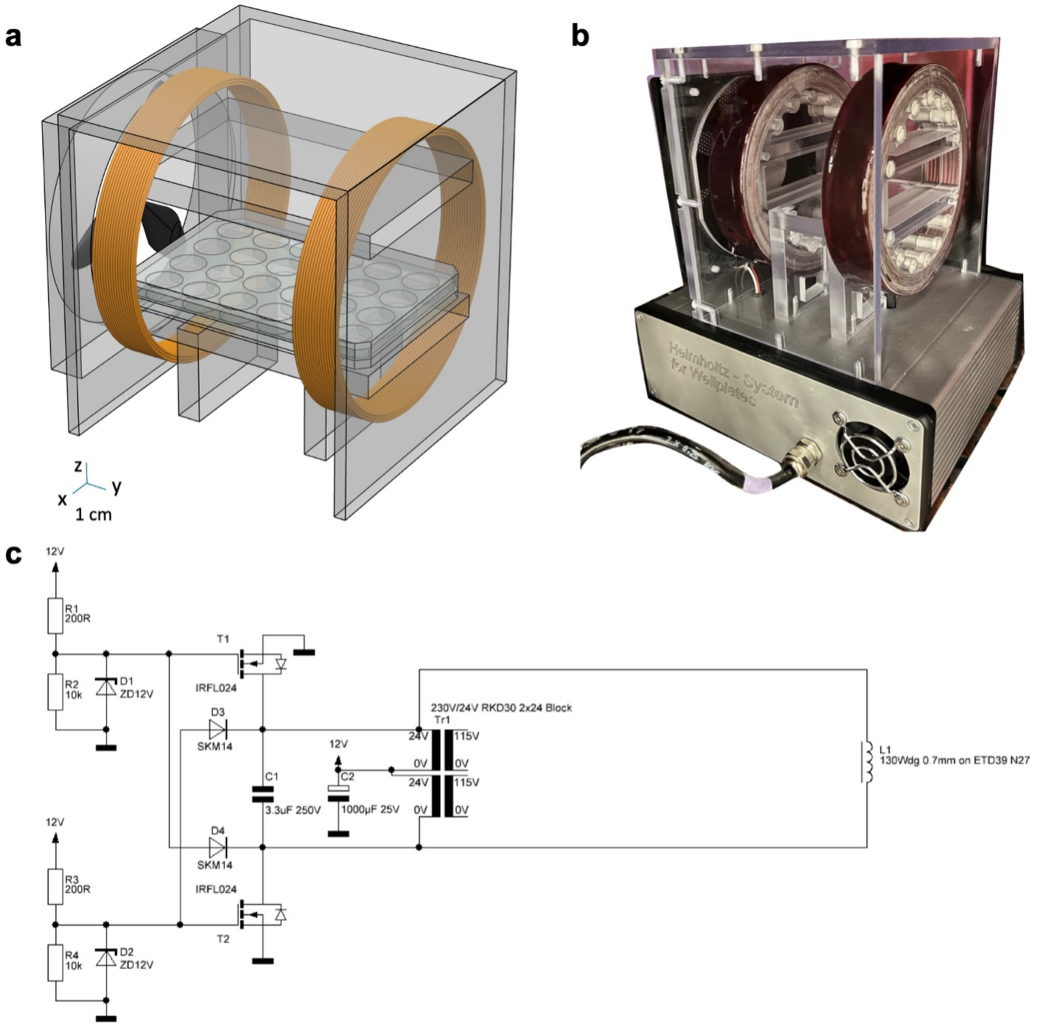

Supplementary Figure 5. Helmholtz-coil-based electromagnetic stimulation device. (**a**) Illustration of the device with scale. Samples were exposed to a uniform EMF by placing them in the central area (5.8 cm x 5.8 cm) of the Helmholtz coil-based device. (**b**) Photo of the device. (**c**) Schematic of the driving circuits of the electromagnetic field device.

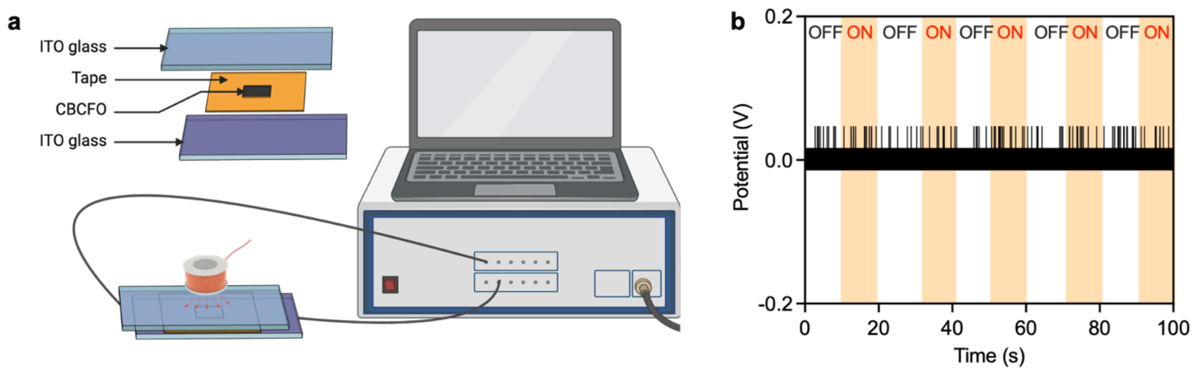

Supplementary Figure 6. Detection of magnetoelectrically driven charge carriers of CBCFO nanoparticles under an alternating current (AC) magnetic field. (a) The experimental setup of open-circuit-voltage (OCV) measurement. CBCFO nanoparticles were placed between the conductive face of two ITO glasses (2×2×1 mm), framed by nonconductive tape, which were connected to an electrochemical station in open-circuit-voltage mode. When the AC EMF (21 mT, 1 kHz) is applied, the induced potential of CBCFO nanoparticles is recorded by the OCV measurement. **(b)** The original circuit bias of the setup. Schematic illustrations of the experimental setup were created with Biorender.com.

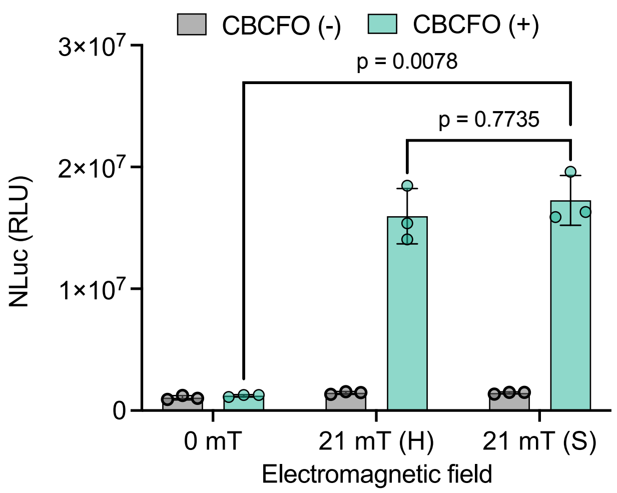

Supplementary Figure 7. NLuc expression of encapsulated $HEK_{EMPOWER}$ cells stimulated by different devices. H refers to the Helmholtz-coil-based device and S refers to the single-coil-based device. Both stimulations were done at 1 kHz, 21 mT for 3 min. The CBCFO concentration was 50 µg/10^6 cells. All data are presented as means ± SD; n = 3 independent experiments. Statistical significances were calculated via two-way ANOVA Turkey's multiple comparison tests.

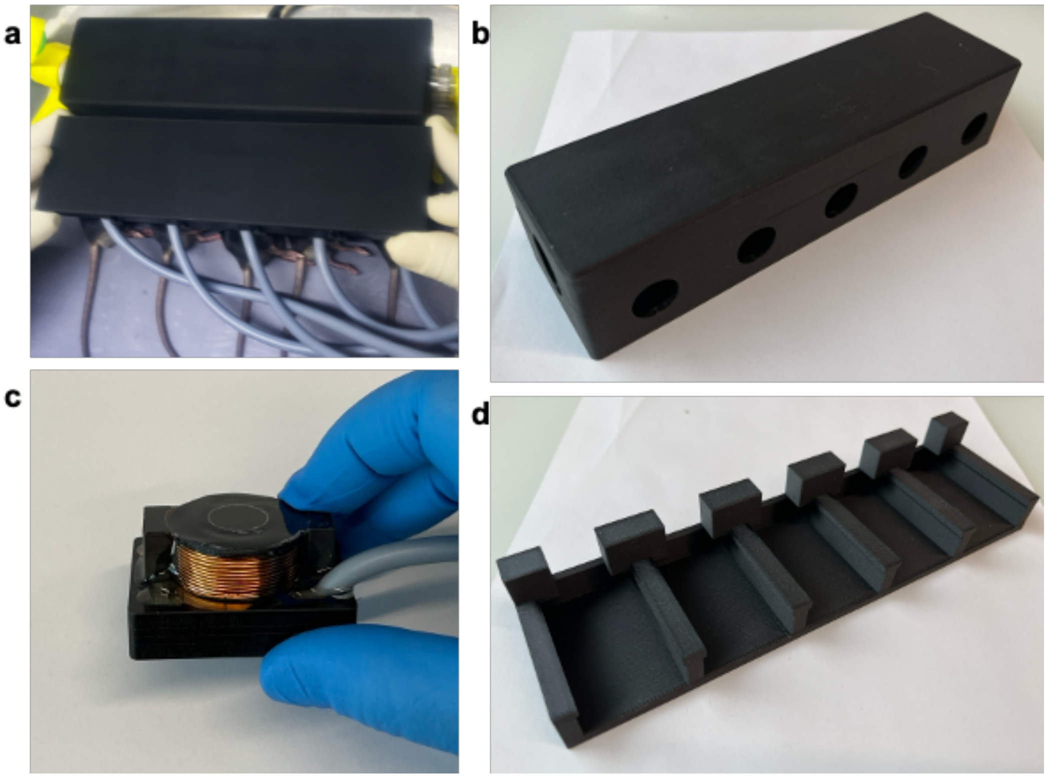

Supplementary Figure 8. Electromagnetic stimulation device based on single-coil magnet for the parallel in vivo experiment. (**a**) Magnetic-field stimulation of 5 mice. (**b**) Isoflurane vent for mice. (**c**) Single-coil electromagnet field device. (**d**) Holder for 5 single-coil devices for parallel experiments in mice.

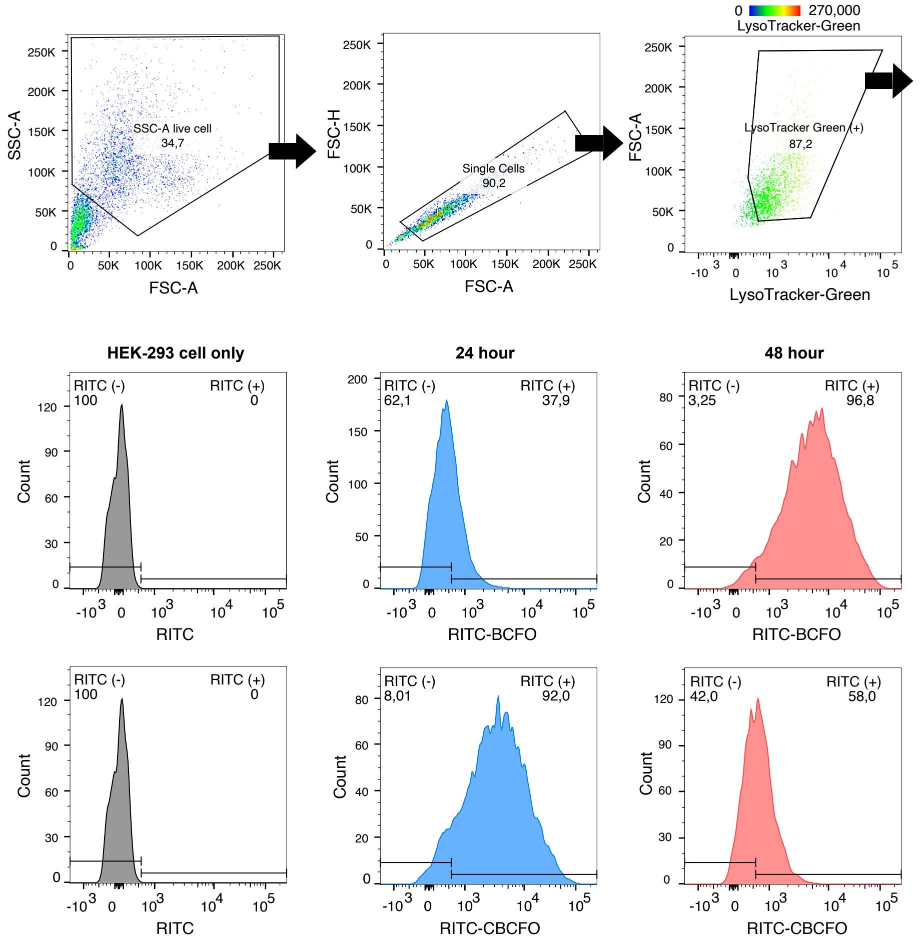

Supplementary Figure 9. Gating strategy and analysis of flowcytometry results in Extended Data Figure 4 f and g.

Supplementary Table 1. Plasmids used and designed in this study

Plasmid	Description	Reference
BB6-BlastR	SB100X-specific transposon containing a constitutive BlastR and iRFP expression unit (ITR-MCS:P_{hCMV}-BlastR-P2A-iRFP-pA-ITR).	Huang et al., 2023.[4]
BB6-PuroR	SB100X-specific transposon containing a constitutive ECFP and PuroR expression unit (ITR-MCS:P_{RPBSA}-ECFP-P2A-PuroR-pA-ITR).	Huang et al., 2023.[4]
BB6-ZeoR	SB100X-specific transposon containing a constitutive ZeoR and mRuby expression unit (ITR-MCS-pA: P_{hCMV}-ZeoR-P2A-mRuby-pA-ITR).	Huang et al., 2023.[4]
pcDNA3.1(+)	Constitutive mammalian expression vector containing a NeoR resistance gene (P_{hCMV}-MCS-pA).	Thermo Fisher Scientific, CA
pJH42	Constitutive SB100X expression vector (P_{hCMV}-SB100X-pA).	Huang et al., 2023.[4]
pJH1003	Constitutive NRF2 expression vector (P_{hCMV}-NRF2-pA).	Huang et al., 2023.[4]
pJH1004	Constitutive KEAP1 expression vector (P_{hCMV}-KEAP1-pA).	Huang et al., 2023.[4]
pJH1005	ARE-driven SEAP expression vector (P_{DART1}-SEAP-pA).	Huang et al., 2023.[4]
pJH1010	Four tandem ARE-driven SEAP expression vector (P_{DART4}-SEAP-pA).	Huang et al., 2023.[4]

pJH1054	SB100X-specific transposon containing a constitutive KEAP1 and BlastR expression unit.<br>(ITR-P_{hCMV}-KEAP1-P2A-BlastR-pA-ITR).	Huang et al., 2023.[4]
pJH1096	SB100X-specific transposon ARE-driven NLuc and mouse insulin expression unit, and a constitutive ZeoR expression unit.<br>(ITR-PDART-NLuc-P2A-mINS: P_{mPGK}-ZeoR-pA- ITR)	Huang et al., 2023.[4]
pJH1101	SB100X-specific transposon containing a constitutive NRF2 expression unit and a constitutive ECFP and PuroR expression unit<br>(ITR-P_{hCMV}-NRF2-pA: P_{RPBSA}-ECFP-P2A-PuroR-pA-ITR).	Huang et al., 2023.[4]

Abbreviations: **ARE**: antioxidant response element; **BlastR**, gene conferring blasticidin resistance; **CMV**, cytomegalovirus; **ECFP**, enhanced cyan fluorescent protein; **iRFP**, near-infrared fluorescent protein; **ITR**, inverted terminal repeats of SB100X; **KEAP1**: Kelch-like ECH-associated protein 1; **P_{mPGK}**: a mouse constitutive promotor; **MCS**, multiple cloning site; **mINS**, modified insulin variant for optimal expression in HEK-293 cells; **mRuby**: a bright monomeric red fluorescent protein; **Nluc**, *Oplophorus gracilirostris* luciferase; **NRF2**: nuclear factor erythroid 2 p45-related factor 2; **P2A**, picornavirus-derived ribosome skipping sequence optimized for bicistronic expression in mammalian cells; **pA**, polyadenylation signal; **PCR**, polymerase chain reaction; **P_{DART}**, promotor containing ARE element, O_{ARE}-$P_{hCMVmin}$; **P_{DART4}**, O_{ARE4}-$P_{hCMVmin}$; **P_{hCMV}**, human cytomegalovirus immediate early promoter; **$P_{hCMVmin}$**, minimal version of P_{hCMV}; **P_{RPBSA}**: a constitutive synthetic mammalian promotor; **PuroR**, gene conferring puromycin resistance; **SB100X**, optimized Sleeping Beauty transposase; **SEAP**, human placental secreted alkaline phosphatase; **ZeoR**, gene conferring zeocin resistance.

Reference

1 Schlatter, S., Rimann, M., Kelm, J. & Fussenegger, M. SAMY , a Novel Mammalian Reporter Gene Derived from Bacillus Stearothermophilus α-amylase. *Gene* **282**, 19-31 (2002).

2 Rennick, J. J., Johnston, A. P. R. & Parton, R. G. Key Principles and Methods for Studying the Endocytosis of Biological and Nanoparticle Therapeutics. *Nat. Nanotech.* **16**, 266-276 (2021).

3 Liu, Y. *et al.* Targeted Protein Degradation via Cellular Trafficking of Nanoparticles. *Nat. Nanotech.* **20**, 296–302 (2025).

4 Huang, J., Xue, S., Buchmann, P., Teixeira, A. P. & Fussenegger, M. An electrogenetic interface to program mammalian gene expression by direct current. *Nat. Metab.* **5**, 1395-1407 (2023).
