# Supplementary material for: Electromagnetic wireless remote control of mammalian transgene expression
Source: Nat Nanotechnol. 2025 May 5;20(8):1071–8. doi: 10.1038/s41565-025-01929-w (PMC12373504; doi:10.1038/s41565-025-01929-w)
Supplement: Supplementary file 2 — Reporting Summary [file 41565_2025_1929_MOESM2_ESM.pdf]

Corresponding author(s): Martin Fussenegger

Last updated by author(s): Mar 28, 2025

Reporting Summary

Nature Portfolio wishes to improve the reproducibility of the work that we publish. This form provides structure for consistency and transparency in reporting. For further information on Nature Portfolio policies, see our Editorial Policies and the Editorial Policy Checklist.

Statistics

For all statistical analyses, confirm that the following items are present in the figure legend, table legend, main text, or Methods section.

n/a	Confirmed	
☐	☒	The exact sample size (*n*) for each experimental group/condition, given as a discrete number and unit of measurement
☐	☒	A statement on whether measurements were taken from distinct samples or whether the same sample was measured repeatedly
☐	☒	The statistical test(s) used AND whether they are one- or two-sided<br>*Only common tests should be described solely by name; describe more complex techniques in the Methods section.*
☐	☒	A description of all covariates tested
☒	☐	A description of any assumptions or corrections, such as tests of normality and adjustment for multiple comparisons
☐	☒	A full description of the statistical parameters including central tendency (e.g. means) or other basic estimates (e.g. regression coefficient) AND variation (e.g. standard deviation) or associated estimates of uncertainty (e.g. confidence intervals)
☐	☒	For null hypothesis testing, the test statistic (e.g. *F*, *t*, *r*) with confidence intervals, effect sizes, degrees of freedom and *P* value noted<br>*Give P values as exact values whenever suitable.*
☒	☐	For Bayesian analysis, information on the choice of priors and Markov chain Monte Carlo settings
☒	☐	For hierarchical and complex designs, identification of the appropriate level for tests and full reporting of outcomes
☒	☐	Estimates of effect sizes (e.g. Cohen's *d*, Pearson's *r*), indicating how they were calculated

Our web collection on statistics for biologists contains articles on many of the points above.

Software and code

Policy information about availability of computer code

Data collection	No code used in this study.
Data analysis	For statistical evaluations involving multiple comparisons, GraphPad Prism 8 (v 9.2.0, GraphPad Software Inc.) and Microsoft Excel (v16.51, Microsoft) were employed. A two-tailed, unpaired Student's t-test and one-way or two-way analysis of variance (ANOVA) were utilized to determine the statistical significance of differences. The images were analyzed and Pearson's correlation coefficients were calculated by FIJI ImageJ with the Colocalization plugin.

For manuscripts utilizing custom algorithms or software that are central to the research but not yet described in published literature, software must be made available to editors and reviewers. We strongly encourage code deposition in a community repository (e.g. GitHub). See the Nature Portfolio guidelines for submitting code & software for further information.

Data

Policy information about availability of data

All manuscripts must include a data availability statement. This statement should provide the following information, where applicable:

- Accession codes, unique identifiers, or web links for publicly available datasets
- A description of any restrictions on data availability
- For clinical datasets or third party data, please ensure that the statement adheres to our policy

The authors declare that all the data supporting the findings of this study are available within the paper and its supplementary materials.

Research involving human participants, their data, or biological material

Policy information about studies with human participants or human data. See also policy information about sex, gender (identity/presentation), and sexual orientation and race, ethnicity and racism.

Reporting on sex and gender	The authors declare that no human research participants were involved in this study.
Reporting on race, ethnicity, or other socially relevant groupings	The authors declare that no human research participants were involved in this study.
Population characteristics	The authors declare that no human research participants were involved in this study.
Recruitment	The authors declare that no human research participants were involved in this study.
Ethics oversight	The authors declare that no human research participants were involved in this study.

Note that full information on the approval of the study protocol must also be provided in the manuscript.

Field-specific reporting

Please select the one below that is the best fit for your research. If you are not sure, read the appropriate sections before making your selection.

☒ Life sciences ☐ Behavioural & social sciences ☐ Ecological, evolutionary & environmental sciences

For a reference copy of the document with all sections, see nature.com/documents/nr-reporting-summary-flat.pdf

Life sciences study design

All studies must disclose on these points even when the disclosure is negative.

Sample size	In practice, the sample size used in this study is usually determined based on the need for it to offer sufficient statistical power, and the time, cost, or convenience of collecting the data. No specific statistical methods were used to predetermine sample size.
Data exclusions	No data were excluded from the analyses.
Replication	All experiments in this study were successfully reproduced at least twice.
Randomization	All samples in this study were allocated randomly.
Blinding	All investigators involved in this study were blinded to group allocation during data collection and analysis.

Reporting for specific materials, systems and methods

We require information from authors about some types of materials, experimental systems and methods used in many studies. Here, indicate whether each material, system or method listed is relevant to your study. If you are not sure if a list item applies to your research, read the appropriate section before selecting a response.

Materials & experimental systems

n/a	Involved in the study	
☐	☒	Antibodies
☐	☒	Eukaryotic cell lines
☒	☐	Palaeontology and archaeology
☐	☒	Animals and other organisms
☒	☐	Clinical data
☒	☐	Dual use research of concern
☒	☐	Plants

Methods

n/a	Involved in the study	
☒	☐	ChIP-seq
☐	☒	Flow cytometry
☒	☐	MRI-based neuroimaging

Antibodies

Antibodies used	Anti-KEAP1, Abcam, Cat. no. ab227828, Western blot (1:5000), Lot. no. GR3397951-8;<br>Anti-NRF2, Abcam, cat. no. ab137550, Western blot (1:5000), Lot. no. GR3419093-1;<br>Anti-vinculin, Cell Signaling, Cat.no. 4650, Western blot (1:1000); lot.no. 5

	Anti-Cytochrome c, Abcam, cat.no. ab65311, Western blot (1:500); lot.no. 1082637-1;<br>Donkey anti-rabbit IgG (secondary), Sigma, cat. no. GENA934, Western blot (1:10000), Lot. no. 17528149;<br>Sheep anti-mouse IgG (secondary), Sigma, cat. no. GENA931V, Western blot (1:10000), Lot. no. 9739640.
Validation	All the commercially available antibodies used in this study were validated by the manufacturers and or previous publications through Western blot.<br>Anti-KEAP1 (https://www.abcam.com/keap1-antibody-epr22664-26-ab227828.html);<br>Anti-NRF2 (https://www.abcam.com/nrf2-antibody-ab137550.html);<br>Anti-vinculin (https://www.cellsignal.com/products/primary-antibodies/vinculin-antibody/4650);<br>Anti-cytochrome c (https://www.abcam.com/en-us/products/assay-kits/cytochrome-c-release-assay-kit-ab65311)<br>Donkey anti-rabbit IgG (https://www.sigmaaldrich.com/CH/en/product/sigma/gena9341ml);<br>Sheep anti-mouse IgG (https://www.sigmaaldrich.com/CH/en/product/sigma/gena9311ml).

Eukaryotic cell lines

Policy information about cell lines and Sex and Gender in Research

Cell line source(s)	Cell lines used in this study: HEK-293, ATCC: CRL-11268; hMSC-TERT (Simonsen et al., Nature Biotechnology, 2002); BHK-21, ATCC: CCL-10; CHO-K1, ATCC: CCL-61; Hep G2, ATCC: CRL-11997; AtT-20, ATCC: CCL-89;
Authentication	All the cell lines used in this study were authenticated by the supplier and the authorities of the Department of Biosystems Science and Engineering (D-BSSE) of the ETH Zurich in Basel, Switzerland.
Mycoplasma contamination	The authors declare that all the cell lines in this study were tested negative for mycoplasma contamination.
Commonly misidentified lines<br>(See ICLAC register)	No commonly misidentified cell lines were used in this study.

Animals and other research organisms

Policy information about studies involving animals; ARRIVE guidelines recommended for reporting animal research, and Sex and Gender in Research

Laboratory animals	The 8-week-old wild-type male Swiss mice (C57BL/6J, Janvier Labs) were used in this study.
Wild animals	The authors declare that no wild animals were used in this study.
Reporting on sex	The male mice used in this study were randomly selected by following previous studies (Bai et al., Nature Medicine, 2019; Krawczyk et al., Science, 2020; Zhou et al., Nature Biotechnology, 2021; Chen et al., Nature Chemical Biology; Schneider et al., Science Advances, 2021, Huang et al., Nature metabolism, 2023), as well as due to the convenience of husbandry with the same sex. Sex was not considered in study design. No data disaggregated for sex were collected.
Field-collected samples	The authors declare that no field-collected samples were used in this study.
Ethics oversight	All procedures were performed in compliance with Swiss animal welfare regulations, approved by the Veterinary Office of the Canton Basel-Stadt, Switzerland (license number: 2996_34477), the French Republic (Project No. DR2018-40v5 and APAFIS No. 16753) and the People's Republic of China (Institutional Animal Care and Use Committee (IACUC) of Westlake University, Protocol ID20-009-XMQ). The experiments were conducted by P.G.R (License number: LTK 5507; Department of Biosystems Science and Engineering (D-BSSE), ETH Zurich, Basel, Switzerland), G. Charpin-El Hamri (No. 69266309; University of Lyon, Institut Universitaire de Technologie) or by S. Xue (Westlake University).

Note that full information on the approval of the study protocol must also be provided in the manuscript.

Plants

Seed stocks	The authors declare that no plant-related research in this study.
Novel plant genotypes	The authors declare that no plant-related research in this study.
Authentication	The authors declare that no plant-related research in this study.

Flow Cytometry

Plots

Confirm that:

☒ The axis labels state the marker and fluorochrome used (e.g. CD4-FITC).

☒ The axis scales are clearly visible. Include numbers along axes only for bottom left plot of group (a 'group' is an analysis of identical markers).

☒ All plots are contour plots with outliers or pseudocolor plots.

☒ A numerical value for number of cells or percentage (with statistics) is provided.

Methodology

Sample preparation	The sample preparation is described in detail in the methods section of the manuscript. Briefly, HEK-293 cells were seeded in 24-well plates (1 x10^5 cells per well), incubated with Rhodamine B isothiocyanate iRll.. 40 uiM, Cat. No.CAY20653-100mg, Cayman)-labelled nanoparticles, and sequentially stained with Lyso-Tracker Green (50 nM, Cat. No. 8783, Cell Signaling) at 37 °C for 1h. HEK-293 cells stained with Lyso-Tracker Green were used as a negative control. These counterstained cells were subjected to flow cytometric analysis.
Instrument	Flow cytometry analysis was performed on a FACSAria Fusion Cell Sorter, Becton Dickinson, New Jersey, USA
Software	Flow cytometry data were analyzed with FlowJo 10.5 software.
Cell population abundance	Using fluorescent output, positive cells for RITC and Lyso-Tracker Green signals were analyzed as described in sample preparation. The results are included in supplementary file for more clarity.
Gating strategy	A comprehensive report is attached in supplementary file. Gating for positive cells was performed based on HEK-293 cells expressing no fluorophore.

☒ Tick this box to confirm that a figure exemplifying the gating strategy is provided in the Supplementary Information.
