# Supplementary figures and images for: Electromagnetic wireless remote control of mammalian transgene expression
Source: Nat Nanotechnol. 2025 May 5;20(8):1071–8. doi: 10.1038/s41565-025-01929-w (PMC12373504; doi:10.1038/s41565-025-01929-w)

# Uncropped western blot images in Extended Data Fig. 1d

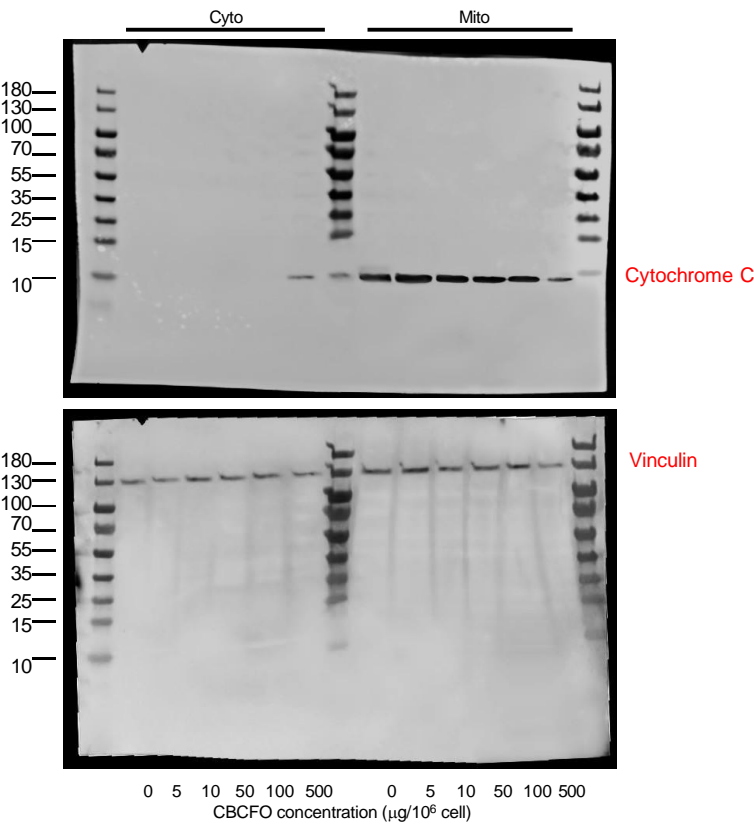

Supplement: Supplementary file 9 — Unprocessed western blots for Extended Data Fig. 1d [file 41565_2025_1929_MOESM9_ESM.pdf]

Uncropped western blot images in Extended Data Fig. 7b

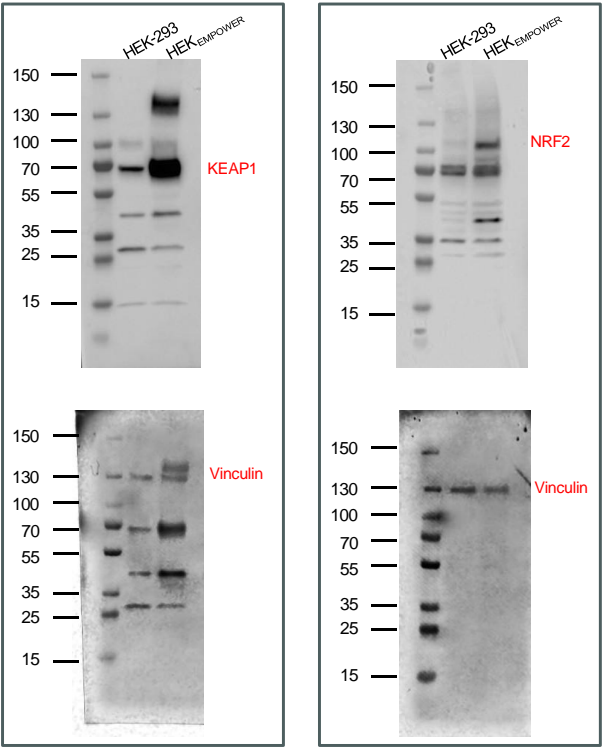

Supplement: Supplementary file 16 — Unprocessed western blots for Extended Data Fig. 7b [file 41565_2025_1929_MOESM16_ESM.pdf]
